# Prediction of Lindane-Induced Breast Cancer Toxicity Through Multi-Target Network Analysis and Mechanistic Investigation

**DOI:** 10.3390/ijms27209056

**Published:** 2026-10-13

**Authors:** Yuze Lin, Jiahui Xu, Mingyang Sheng, Zidong Yang, Jiatong Liu, Xiangze Fan, Lin Wang, Wei Xu

**Affiliations:** 1 College of Pharmacy, Changchun University of Chinese Medicine, Changchun 130117, China; 2 College of Innovation and Entrepreneurship, Changchun University of Chinese Medicine, Changchun 130117, China

**Keywords:** breast cancer, Lindane, oxidative stress, NR3C2, network toxicology, machine learning, SHAP analysis

## Abstract

This study aimed to identify potential molecular targets associated with Lindane-induced molecular alterations in breast cancer and elucidate its toxicological mechanisms using an integrated strategy combining network toxicology, machine learning, molecular simulation, and experimental validation. Lindane-related targets and breast cancer transcriptomic data were collected from public databases, and differentially expressed genes and WGCNA-derived hub genes were integrated to identify candidate targets. GO and KEGG analyses were performed to explore biological functions, while machine learning models and SHAP analysis were applied to screen potential biomarkers. Molecular docking and 100 ns molecular dynamics simulations were conducted to evaluate the interaction between Lindane and key proteins. Twenty-eight overlapping targets were identified and mainly enriched in lipid metabolism, sphingolipid signaling, and p53-related pathways. The LASSO-plsRglm model achieved the highest average AUC (0.996) and identified five candidate genes, including NR3C2, AR, TGM2, ATAD2, and P2RY2. NR3C2 exhibited strong discriminatory performance (AUC = 0.982) and was consistently downregulated in breast cancer tissues. Molecular simulations suggested a potential interaction between Lindane and NR3C2. Cell experiments using human breast cancer MCF-7 cells demonstrated that Lindane increased intracellular ROS accumulation and promoted apoptosis, accompanied by decreased NR3C2 expression, upregulation of TGM2 and ATAD2, increased p53, Bax, and cleaved caspase-3 protein levels, and reduced Bcl-2 expression. NR3C2 may serve as a potential regulatory molecule involved in cellular responses to Lindane exposure in breast cancer, providing new insights into the molecular mechanisms underlying environmental Lindane exposure and breast cancer-related toxicity.

## 1. Introduction

Breast cancer (BC) is one of the most frequently diagnosed malignant tumors among women worldwide and remains a leading cause of cancer-related mortality [1,2]. According to global cancer statistics, the burden of breast cancer continues to increase, with its incidence and mortality influenced by multiple factors, including population aging, lifestyle changes, and environmental exposures [3]. Although substantial progress has been achieved in early screening, molecular classification, and precision therapies, some patients still exhibit limited treatment responses, high recurrence rates, and unfavorable clinical outcomes [4]. Therefore, identifying potential risk factors associated with breast cancer development, particularly the molecular mechanisms underlying environmental exposure-related effects, is critical for disease prevention and targeted intervention.

Breast cancer is a multifactorial disease resulting from complex interactions among genetic, hormonal, environmental, and biological factors [5]. Its initiation and progression involve diverse molecular processes, including genetic alterations, endocrine disruption, chronic inflammation, oxidative stress imbalance, and immune microenvironment remodeling [6]. Among environmental factors, endocrine-disrupting chemicals (EDCs) have attracted considerable attention due to their ability to mimic or interfere with endogenous hormone signaling and their potential involvement in hormone-related diseases [7,8]. Increasing evidence suggests that long-term exposure to environmental pollutants may promote abnormal breast tissue proliferation and malignant transformation by modulating hormone receptor activity, regulating cellular proliferation, and disrupting redox homeostasis [9]. However, the molecular networks and regulatory pathways linking environmental pollutant exposure to breast cancer remain largely unclear.

Lindane (C_6_H_6_Cl_6_), a representative organochlorine pesticide, was widely used in agricultural and public health applications due to its potent insecticidal activity. However, its chemical stability, slow environmental degradation, and high lipid solubility contribute to its persistence in ecosystems and bioaccumulation through food chains [10]. Following human exposure, Lindane can accumulate in adipose tissues and may exert adverse biological effects by disrupting endocrine regulation, oxidative balance, and cellular signaling pathways [11].

Previous studies have reported that Lindane exposure can induce reactive oxygen species (ROS) accumulation and oxidative stress, potentially resulting in genomic instability, altered cell cycle regulation, and abnormal apoptotic responses [12]. As a potential endocrine-disrupting chemical, Lindane may also influence hormone-dependent diseases through interference with hormone receptor-mediated signaling pathways [13]. Nevertheless, current investigations into the relationship between Lindane exposure and breast cancer have mainly focused on general toxicological outcomes, whereas the specific molecular targets, regulatory networks, and biological mechanisms remain insufficiently characterized.

Recent advances in high-throughput sequencing technologies, public databases, and computational approaches have provided new opportunities to investigate the biological effects of environmental chemicals. The integration of network toxicology, bioinformatics, functional enrichment analysis, and machine learning algorithms enables the systematic identification of chemical-associated targets, disease-related genes, and potential regulatory molecules within complex biological networks [14]. Furthermore, interpretable machine learning approaches, such as SHAP (Shapley Additive Explanations) analysis, can quantify the contribution of individual features to model predictions, thereby improving model interpretability and reliability.

Therefore, this study established an integrated framework combining network toxicology, bioinformatics analysis, machine learning, molecular simulation, and experimental validation to investigate the molecular mechanisms underlying Lindane-associated molecular alterations in breast cancer [15]. Potential Lindane targets were obtained through database mining, and breast cancer-associated genes were identified through transcriptomic analysis. Subsequently, machine learning models were applied to screen potential biomarkers, and SHAP analysis was performed to interpret their contributions to model predictions [16]. Immune infiltration analysis, TCGA database validation, molecular docking, and molecular dynamics simulations were further conducted to explore potential regulatory mechanisms [17,18]. Finally, cell-based experiments were performed to validate the effects of Lindane exposure on cell and associated molecular alterations [19].

This study aimed to systematically characterize the molecular network associated with Lindane exposure and breast cancer, identify potential regulatory targets, and provide new insights into the risk assessment and mechanistic understanding of environmental pollutant-associated toxicity.

## 2. Results

### 2.1. Identification of Potential Lindane-Associated Targets

To explore the potential molecular basis underlying the involvement of Lindane in breast cancer, target prediction analysis was initially performed using multiple public databases. Potential targets of Lindane were collected from the ChEMBL, STITCH, SuperPred, and Swiss Target Prediction databases, and the predicted targets from different databases were integrated and standardized. After removing duplicate entries, a total of 145 unique Lindane targets were obtained. These identified targets constituted the Lindane target pool and provided the foundation for subsequent identification of Lindane–breast cancer overlapping targets and functional analyses. The experimental data are provided in the Supplementary Materials.

### 2.2. Integration of GEO Datasets and Identification of Differentially Expressed Genes in Breast Cancer

To identify differentially expressed genes (DEGs) between breast cancer and normal tissues, the GSE22820 and GSE26304 datasets were integrated to generate the training cohort. Since potential batch effects caused by differences in experimental platforms may exist among datasets, the SVA algorithm was applied for batch correction before downstream analyses.

**Figure 1:**
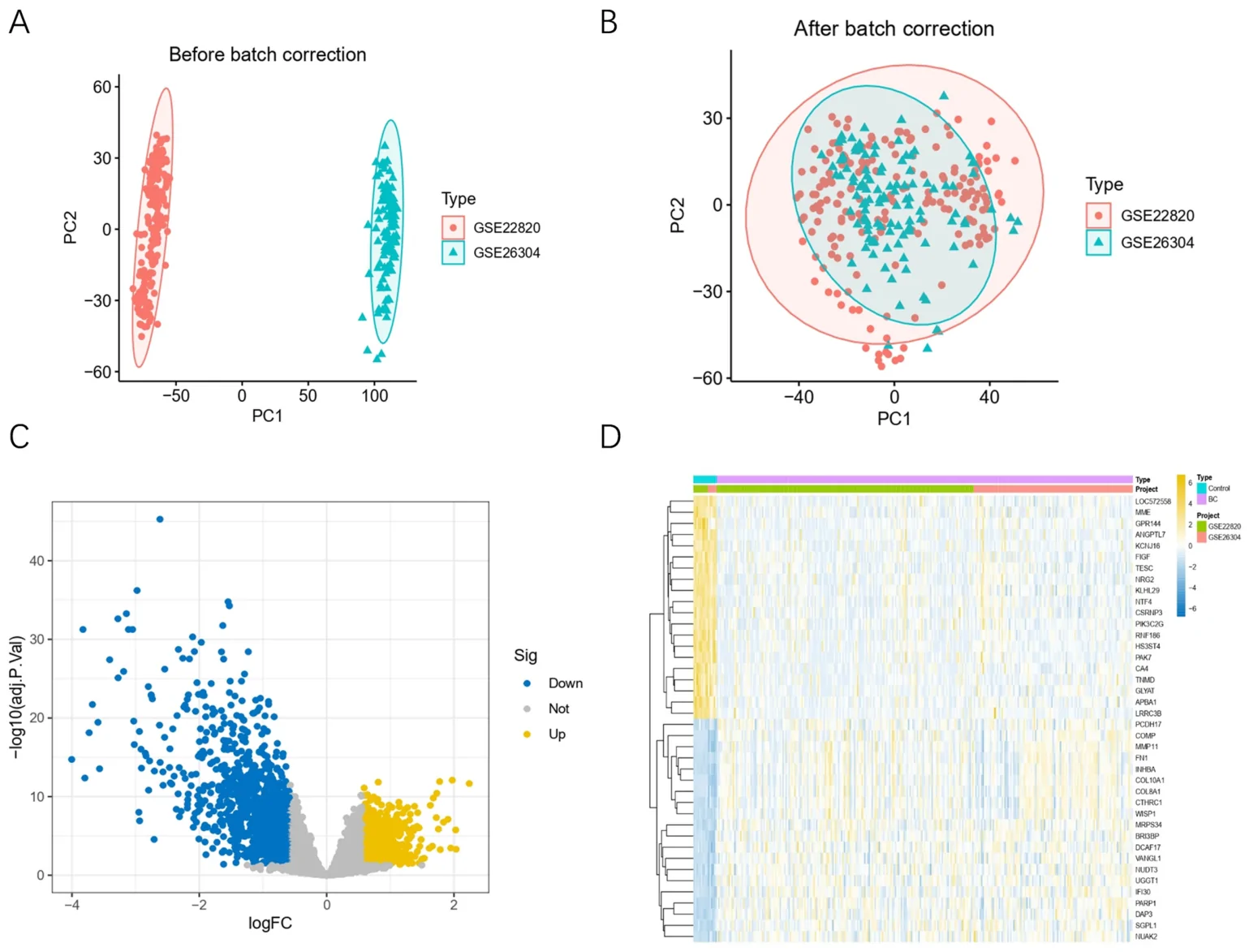
Identification of differentially expressed genes in breast cancer after GEO data integration. (**A**) Batch effect distribution before SVA correction between GSE22820 and GSE26304 datasets. (**B**) Batch effect distribution after SVA correction. (**C**) Volcano plot showing differentially expressed genes between breast cancer and normal samples. Yellow dots represent upregulated genes, blue dots represent downregulated genes, and gray dots represent genes without significant differences. (**D**) Heatmap showing representative differentially expressed genes.

Figure 1A,B show the sample distributions before and after batch correction. Before correction, samples from the two datasets exhibited distinct distribution patterns, whereas samples from different datasets showed more comparable distributions after correction, indicating that potential batch effects were substantially reduced. The integrated training cohort consisted of 285 breast cancer samples and 16 normal breast tissue samples.

Differential expression analysis was performed using the limma package. Based on the criteria of |log_2_FC| > 0.585 and adjusted *p*-value < 0.05, a total of 2108 DEGs were identified, including 822 upregulated genes and 1286 downregulated genes in breast cancer tissues compared with normal tissues.

The volcano plot illustrated the overall distribution of DEGs between breast cancer and normal tissues (Figure 1C), while the heatmap demonstrated the expression patterns of representative DEGs across the two groups (Figure 1D). These results revealed distinct transcriptional profiles between breast cancer and normal tissues, and the identified DEGs were further analyzed for subsequent core gene screening and functional characterization.

### 2.3. WGCNA Identifies Key Gene Modules Associated with Breast Cancer

**Figure 2:**
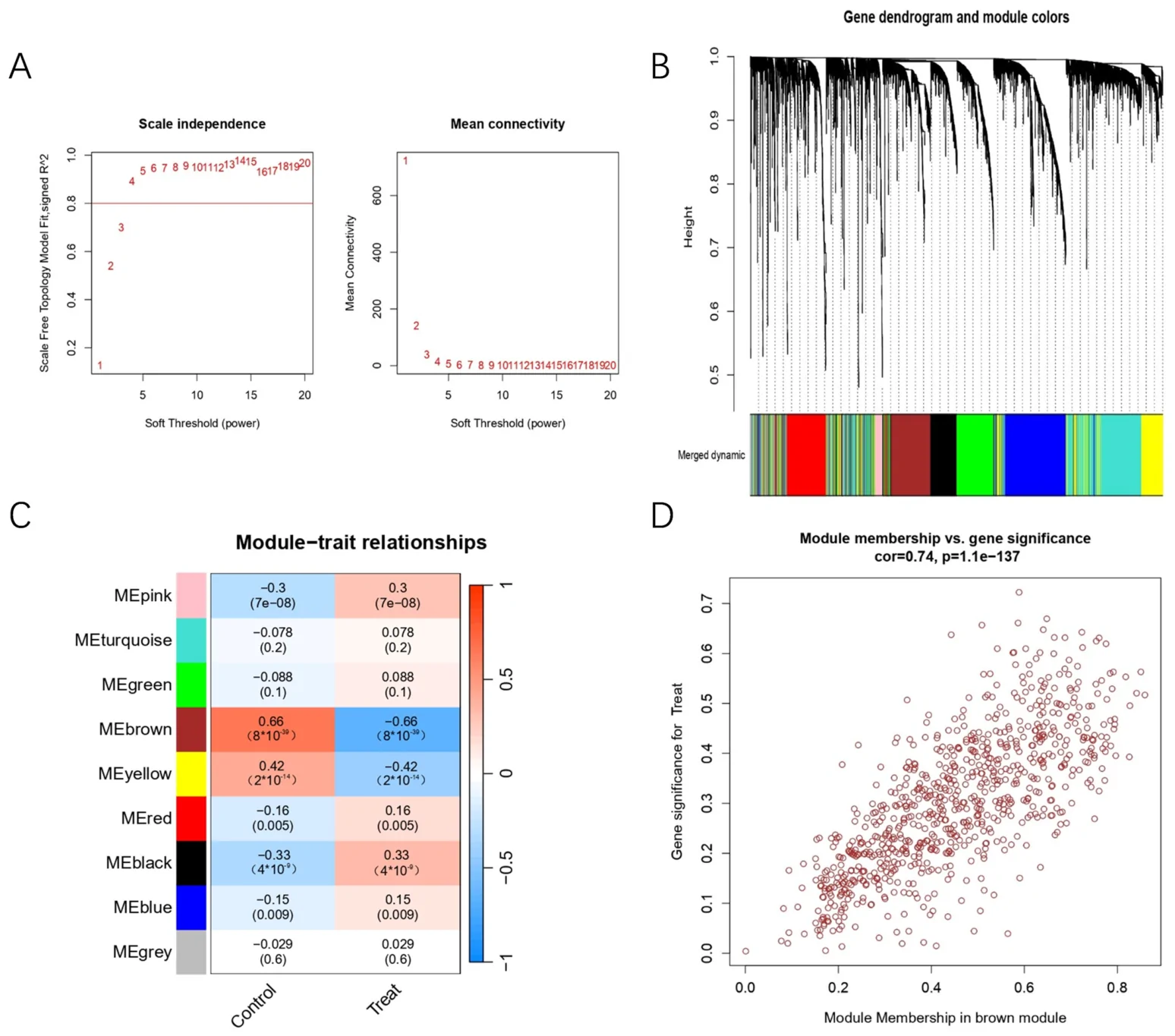
Identification of breast cancer-associated gene modules using WGCNA. (**A**) Analysis of soft-threshold power selection for constructing a scale-free gene co-expression network. (**B**) Hierarchical clustering dendrogram and module identification based on topological overlap matrix. (**C**) Module-trait correlation heatmap showing relationships between gene modules and breast cancer phenotype. (**D**) Gene significance and module membership analysis of the key module.

To identify co-expressed gene modules associated with breast cancer traits, weighted gene co-expression network analysis (WGCNA) was performed using the integrated expression matrix. A soft-thresholding power of β = 4 was selected to achieve scale-free topology with a scale-free topology fit index (R^2^) of 0.85, and the gene co-expression network was subsequently constructed (Figure 2A).

Hierarchical clustering was performed based on the topological overlap matrix (TOM), and the dynamic tree-cut algorithm was applied to identify co-expression modules. Each module was assigned a unique color for visualization (Figure 2B). The relationships between module eigengenes and breast cancer traits were evaluated, and the results were visualized using a module–trait relationship heatmap (Figure 2C).

Among all identified modules, the brown module showed the strongest correlation with breast cancer traits. Therefore, the brown module was identified as the key breast cancer-associated module, and the genes in this module were extracted for subsequent analyses (Figure 2C,D).

### 2.4. Identification of Lindane–Breast Cancer Shared Targets

#### 2.4.1. Integration of Breast Cancer-Related Candidate Targets

Breast cancer-associated candidate genes were identified by integrating DEGs with genes within the key WGCNA module. After merging the two gene sets and removing duplicate genes, a total of 2323 non-redundant breast cancer-associated candidate genes were obtained.

**Figure 3:**
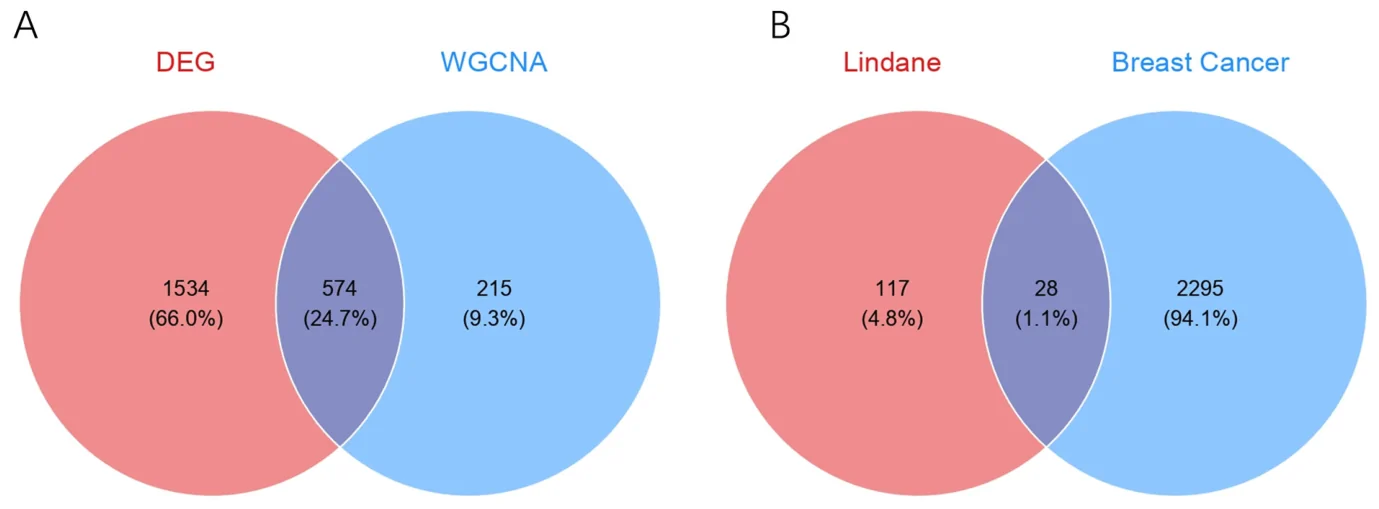
Identification of Lindane-associated breast cancer candidate targets. (**A**) Overlap analysis between differentially expressed genes and WGCNA-derived key module genes. (**B**) Venn diagram showing the intersection between Lindane-related targets and breast cancer-associated genes. The overlapping genes represent potential candidate targets involved in Lindane-associated breast cancer regulation.

The Venn diagram illustrated the intersection between DEGs and WGCNA module genes (Figure 3A). These breast cancer-associated candidate genes were subsequently compared with Lindane targets to identify overlapping targets between Lindane and breast cancer.

#### 2.4.2. Identification of Lindane–Breast Cancer Overlapping Targets

A total of 145 predicted Lindane targets were compared with 2323 breast cancer-associated candidate genes, resulting in 28 overlapping targets. These overlapping targets were considered candidate targets for Lindane-associated breast cancer mechanisms and were subjected to subsequent functional enrichment analysis and machine learning-based screening. The Venn diagram illustrated the intersection between Lindane targets and breast cancer-associated candidate genes (Figure 3B).

### 2.5. GO and KEGG Enrichment Analysis of Lindane–Breast Cancer Overlapping Targets

GO and KEGG enrichment analyses were performed to investigate the potential biological functions and pathways associated with the 28 Lindane–breast cancer overlapping targets.

The GO biological process (BP) analysis revealed that the enriched terms were mainly associated with lipid catabolic processes, cellular ketone metabolism, transport of organic hydroxy compounds, and regulation of lipid metabolism. These results indicated that the Lindane–breast cancer overlapping targets were primarily involved in lipid metabolism-related biological processes.

The cellular component (CC) analysis showed that the enriched terms were mainly associated with the mitochondrial outer membrane, organelle outer membrane, and outer membrane, indicating that the overlapping targets were mainly enriched in mitochondrial membrane-related cellular components.

**Figure 4:**
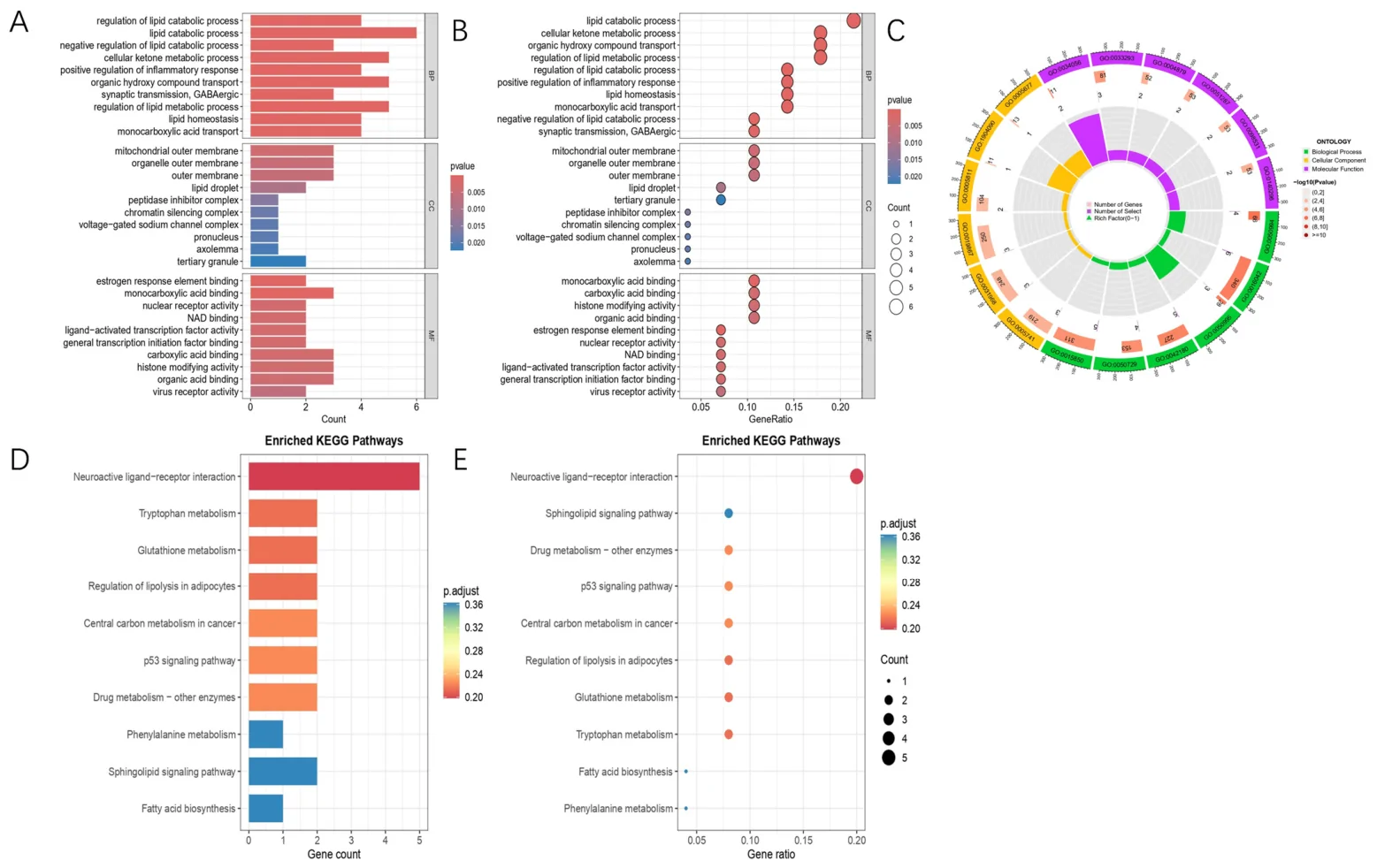
Functional enrichment analysis of Lindane-associated breast cancer targets. (**A**) Bar plot of Gene Ontology (GO) enrichment analysis showing representative terms in biological process (BP), cellular component (CC), and molecular function (MF). The *x*-axis indicates the number of enriched genes (Count), and the color scale represents the enrichment significance. (**B**) Bubble plot of GO enrichment analysis. The *x*-axis represents the Gene Ratio, bubble size indicates the number of enriched genes, and bubble color reflects the enrichment significance. (**C**) Circular plot summarizing the GO enrichment results across BP, CC, and MF categories, including the number of genes, number of selected genes, and enrichment factor. (**D**) Bar plot of Kyoto Encyclopedia of Genes and Genomes (KEGG) pathway enrichment analysis. The *x*-axis indicates the number of enriched genes, and the color scale represents the adjusted *p* value. (**E**) Bubble plot of KEGG pathway enrichment analysis. The *x*-axis represents the Gene Ratio, bubble size indicates the number of enriched genes, and bubble color reflects the adjusted *p* value.

The molecular function (MF) analysis demonstrated that the enriched terms were mainly related to monocarboxylic acid binding, carboxylic acid binding, histone-modifying activity, and organic acid binding. These findings indicated that the Lindane–breast cancer overlapping targets were associated with molecular functions related to metabolite binding and protein modification (Figure 4A–C).

KEGG pathway enrichment analysis revealed that the sphingolipid signaling pathway and p53 signaling pathway were among the significantly enriched pathways. These findings indicated that the Lindane–breast cancer overlapping targets were mainly associated with lipid metabolism, stress response, and apoptosis-related pathways (Figure 4D,E). The common targets were mainly associated with lipid catabolic processes, cellular ketone metabolic processes, mitochondrial outer membrane-related components, and organic acid binding, and were enriched in pathways including neuroactive ligand–receptor interaction, sphingolipid signaling, p53 signaling, and regulation of lipolysis in adipocytes.

### 2.6. Machine Learning-Based Identification of Core Genes and Predictive Model Construction

A multi-algorithm machine learning strategy was applied to identify core genes from the Lindane–breast cancer overlapping targets and construct diagnostic models. 

**Figure 5:**
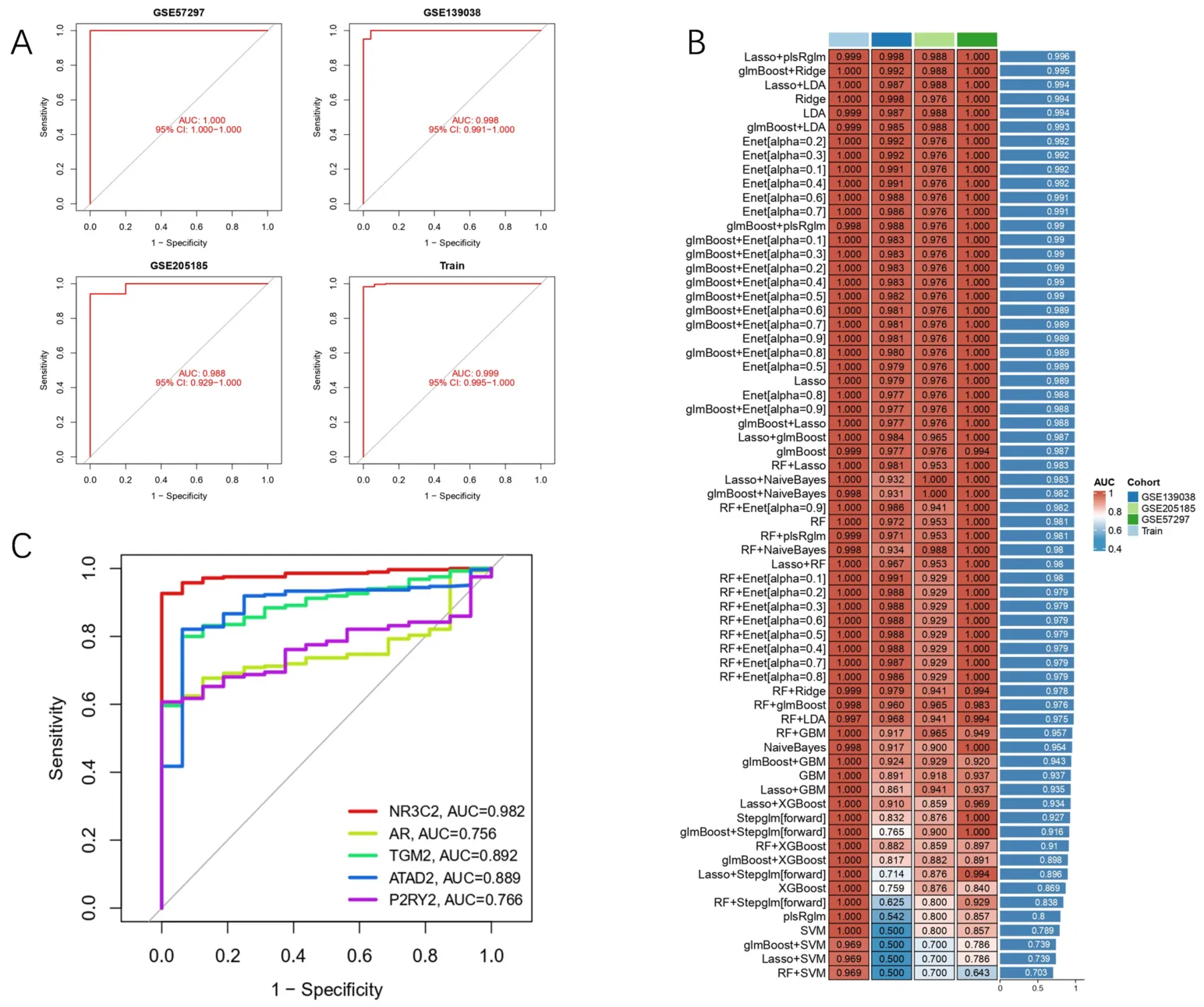
Construction and evaluation of the machine learning-based classification model and identification of core genes. (**A**) Receiver operating characteristic (ROC) curves of the optimal model in the training cohort and three independent validation cohorts, including GSE57297, GSE139038, and GSE205185. The area under the curve (AUC) and corresponding 95% confidence interval are shown in each panel. (**B**) Heatmap comparing the predictive performance of 113 machine learning models across the training and validation cohorts. The color scale represents the AUC value, with warmer colors indicating better classification performance. The LASSO + plsRglm model achieved the highest overall performance across the evaluated datasets. (**C**) ROC curves of the five core genes identified by the optimal model. NR3C2 exhibited the highest single-gene classification performance (AUC = 0.982), followed by TGM2 (AUC = 0.892), ATAD2 (AUC = 0.889), P2RY2 (AUC = 0.766), and AR (AUC = 0.756). The gray diagonal line in the receiver operating characteristic (ROC) curve represents the performance of a random classifier (AUC = 0.5), indicating no discriminatory ability. ROC curves above this reference line indicate better-than-random classification performance, with curves closer to the upper-left corner and higher AUC values reflecting stronger discriminatory ability.

A total of 12 machine learning algorithms were included based on the training cohort data. Different combinations of algorithms were used to construct 113 predictive models. A 10-fold cross-validation strategy was applied to evaluate model performance and stability. The area under the receiver operating characteristic curve (AUC) values were calculated for both the training cohort and the independent validation cohort (Figure 5A).

Figure 5B shows that different machine learning models exhibited varied predictive performance. Among these models, the LASSO + plsRglm model achieved the highest average AUC (0.996), indicating superior diagnostic performance compared with the other models. 

The LASSO + plsRglm model identified five core genes with potential diagnostic value, including nuclear receptor subfamily 3 group C member 2 (NR3C2), androgen receptor (AR), transglutaminase 2 (TGM2), ATPase family AAA domain-containing protein 2 (ATAD2), and purinergic receptor P2Y2 (P2RY2). 

The individual diagnostic performance of the five core genes was further evaluated. NR3C2, TGM2, ATAD2, P2RY2, and AR achieved AUC values of 0.982, 0.892, 0.889, 0.766, and 0.756, respectively (Figure 5C). Among these genes, ATAD2 showed the highest individual diagnostic performance, followed by NR3C2. These findings indicated that the five core genes exhibited potential diagnostic value for distinguishing breast cancer samples from normal tissues.

### 2.7. SHAP-Based Interpretation of Core Gene Contributions

Machine learning models often have complex structures, making their decision-making processes difficult to interpret. Therefore, the SHAP method was applied to quantify feature importance and improve model interpretability.

**Figure 6:**
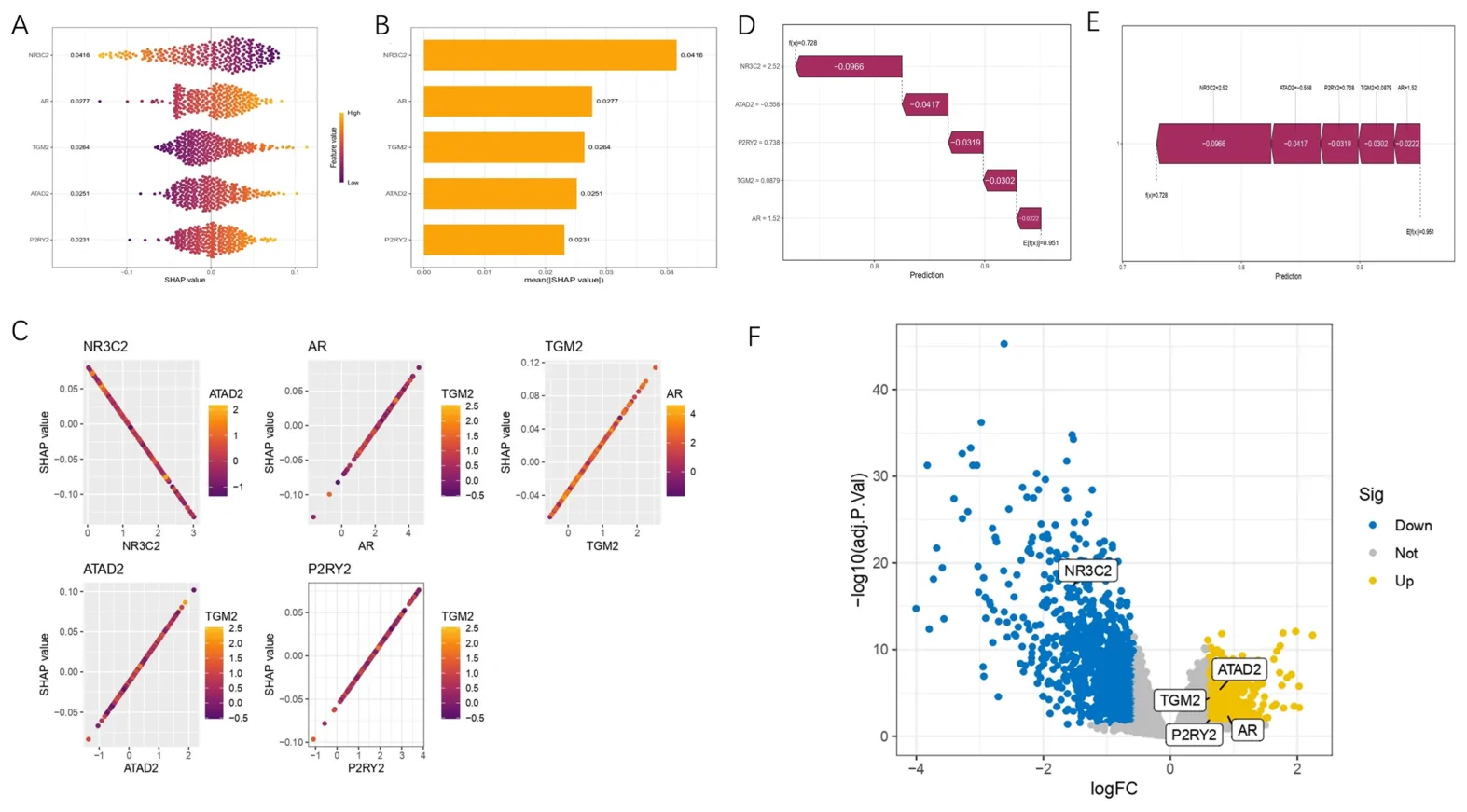
SHAP-based interpretation of the optimal machine learning model and expression patterns of the core genes. (**A**) SHAP summary plot showing the distribution of SHAP values for the five core genes. Each point represents one sample, and the color gradient indicates the corresponding gene expression level, from low to high. The horizontal position reflects the direction and magnitude of each gene’s contribution to the model output. (**B**) Feature importance ranking based on the mean absolute SHAP value. Longer bars indicate a greater overall contribution to model prediction. NR3C2 showed the highest feature importance, followed by AR, TGM2, ATAD2, and P2RY2. (**C**) SHAP dependence plots illustrating the relationships between the expression levels of NR3C2, AR, TGM2, ATAD2, and P2RY2 and their corresponding SHAP values. The color scale represents the expression level of the interacting feature indicated in each panel. (**D**) SHAP waterfall plot for a representative sample, displaying the direction and magnitude of the contribution of each core gene to the final model prediction. (**E**) SHAP force plot for a representative sample, showing the cumulative contributions of individual genes that shifted the model output from the baseline value to the final prediction. (**F**) Volcano plot showing the differential expression of genes between breast cancer and normal samples. Blue, gray, and yellow dots represent downregulated, non-significant, and upregulated genes, respectively. The five machine learning-derived core genes are highlighted and labeled.

The SHAP summary plot was first used to evaluate the overall importance of the five core genes in model predictions. The results showed that NR3C2, AR, and TGM2 exhibited relatively high contributions to the model output (Figure 6A). The SHAP mean importance plot further demonstrated that NR3C2, AR, and TGM2 were major contributing features among the five core genes (Figure 6B).

SHAP dependency plots were further applied to investigate the relationship between gene expression levels and model predictions (Figure 6C). The results showed that variations in gene expression levels were associated with different patterns of feature effects on model predictions. These findings indicated that the five core genes collectively contributed to the predictive performance of the machine learning model.

The SHAP waterfall plots and force plots illustrated the effects of individual features on predictions for specific samples (Figure 6D,E). These visualizations provided further insights into the direction and magnitude of feature impacts on individual model outputs.

By integrating machine learning results with SHAP interpretation, NR3C2 was identified as the most influential feature in the model. Further analysis showed that NR3C2 expression was significantly lower in breast cancer tissues than in normal tissues (Figure 6F). These findings suggested that NR3C2 may represent an important molecular feature associated with breast cancer classification.

### 2.8. Association Between Core Genes and the Breast Cancer Immune Microenvironment

The CIBERSORT algorithm was applied to characterize the immune infiltration landscape and investigate the associations between core genes and immune infiltration patterns in breast cancer. The proportions of 22 immune cell subsets were estimated in the training cohort.

**Figure 7:**
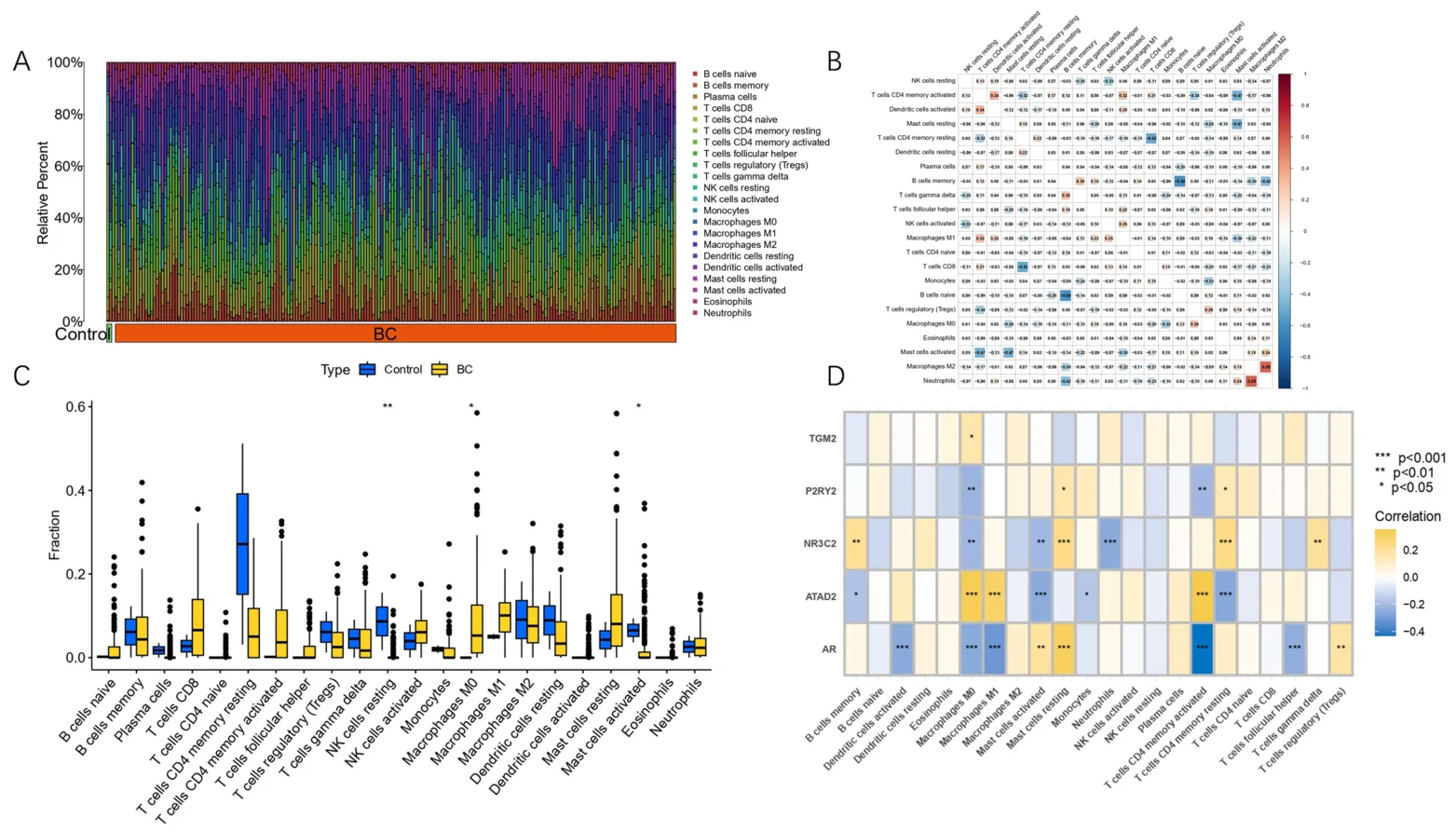
Immune infiltration analysis and correlation between core genes and immune cells. (**A**) Relative proportions of 22 immune cell populations estimated by CIBERSORT. (**B**) Correlation heatmap showing relationships among immune cell subsets. (**C**) Differential immune cell infiltration between breast cancer and normal samples. (**D**) Spearman correlation analysis between core genes and immune cell infiltration levels.

The relative proportions of immune cells in each sample were calculated, and the immune infiltration landscape across samples was visualized using stacked bar charts (Figure 7A). Correlations among different immune cell subsets were further evaluated using an immune cell correlation heatmap (Figure 7B).

Differences in immune cell infiltration abundance between breast cancer and normal tissue groups were further assessed. Several immune cell subsets exhibited significant differences in infiltration abundance between the two groups (Figure 7C).

Spearman correlation analysis was performed to evaluate the associations between the five core genes and immune cell infiltration levels (Figure 7D). The results showed that the expression levels of NR3C2, AR, TGM2, ATAD2, and P2RY2 were significantly correlated with the infiltration levels of specific immune cell subsets.

Among the immune cell subsets significantly associated with core gene expression (*p* < 0.05), M0 macrophages, activated mast cells, and activated CD4 memory T cells showed predominantly negative correlations with core gene expression. In contrast, resting mast cells and resting CD4 memory T cells exhibited predominantly positive correlations. These findings indicated that core gene expression was associated with distinct immune infiltration profiles in breast cancer.

### 2.9. Single-Gene GSVA Reveals Functional Pathways Associated with Core Genes

To investigate the potential biological functions of the core genes, single-gene GSVA was performed based on the expression profiles of NR3C2, AR, TGM2, ATAD2, and P2RY2.

**Figure 8:**
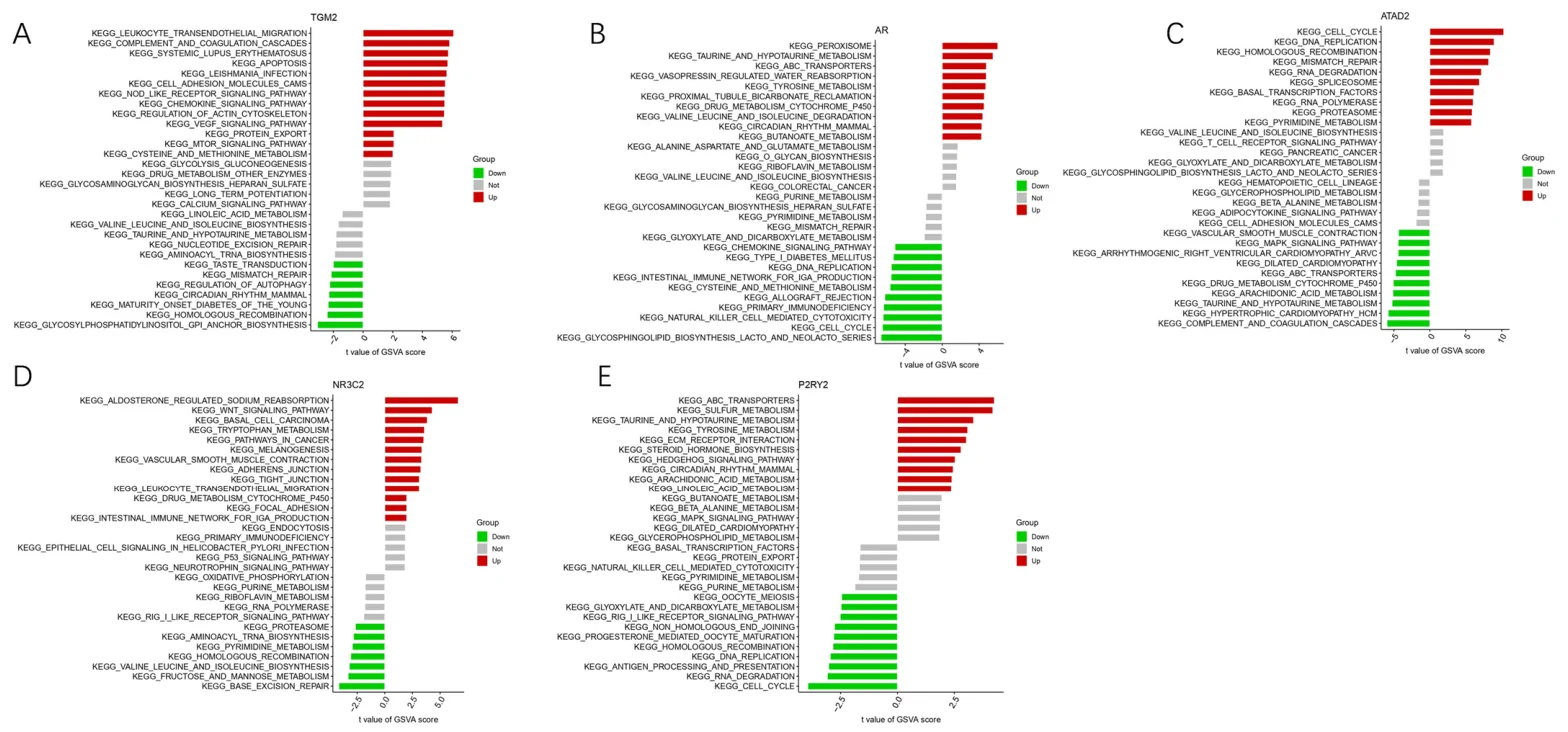
Single-gene GSVA of core genes. (**A**) Pathway enrichment analysis associated with NR3C2 expression. (**B**) Pathway enrichment analysis associated with AR expression. (**C**) Pathway enrichment analysis associated with TGM2 expression. (**D**) Pathway enrichment analysis associated with ATAD2 expression. (**E**) Pathway enrichment analysis associated with P2RY2 expression.

NR3C2 expression was negatively associated with enrichment scores for pathways related to base excision repair, fructose and mannose metabolism, valine, leucine and isoleucine biosynthesis, and homologous recombination (Figure 8A), indicating that NR3C2 may be involved in metabolic regulation and DNA repair-related processes in breast cancer.

AR expression was associated with changes in enrichment scores for pathways related to glycosphingolipid biosynthesis (lacto and neolacto series), cell cycle regulation, natural killer cell-mediated cytotoxicity, and primary immunodeficiency (Figure 8B), suggesting potential involvement in immune regulation, proliferation, and metabolic processes.

TGM2 expression was associated with pathways including glycosylphosphatidylinositol (GPI)-anchor biosynthesis, homologous recombination, maturity-onset diabetes of the young, and circadian rhythm (Figure 8C). These results indicated potential involvement of TGM2 in DNA repair, protein modification, and cellular homeostasis.

ATAD2 expression was associated with complement and coagulation cascades, hypertrophic cardiomyopathy (HCM), taurine and hypotaurine metabolism, and arachidonic acid metabolism pathways (Figure 8D), suggesting associations with inflammatory responses and metabolic regulation.

P2RY2 expression was associated with pathways related to cell cycle regulation, RNA degradation, antigen processing and presentation, and DNA replication (Figure 8E), indicating potential involvement in cellular proliferation, genome maintenance, and immune-related processes.

Collectively, single-gene GSVA demonstrated that the five core genes were associated with diverse biological pathways involving metabolism, DNA repair, cell cycle regulation, and immune responses. These findings provided additional functional insights into the potential biological mechanisms involved in Lindane-associated breast cancer.

### 2.10. Validation of Core Gene Expression Profiles in the TCGA-BRCA Cohort

To further validate the expression patterns of the core genes identified through machine learning in breast cancer, an external validation analysis was performed using the TCGA-BRCA cohort. A total of 1376 samples were included, consisting of 1085 breast cancer tissues and 291 normal breast tissues. The expression levels of the five core genes were compared between tumor and normal tissues to determine whether their expression patterns were consistent with those observed in the GEO cohorts.

**Figure 9:**
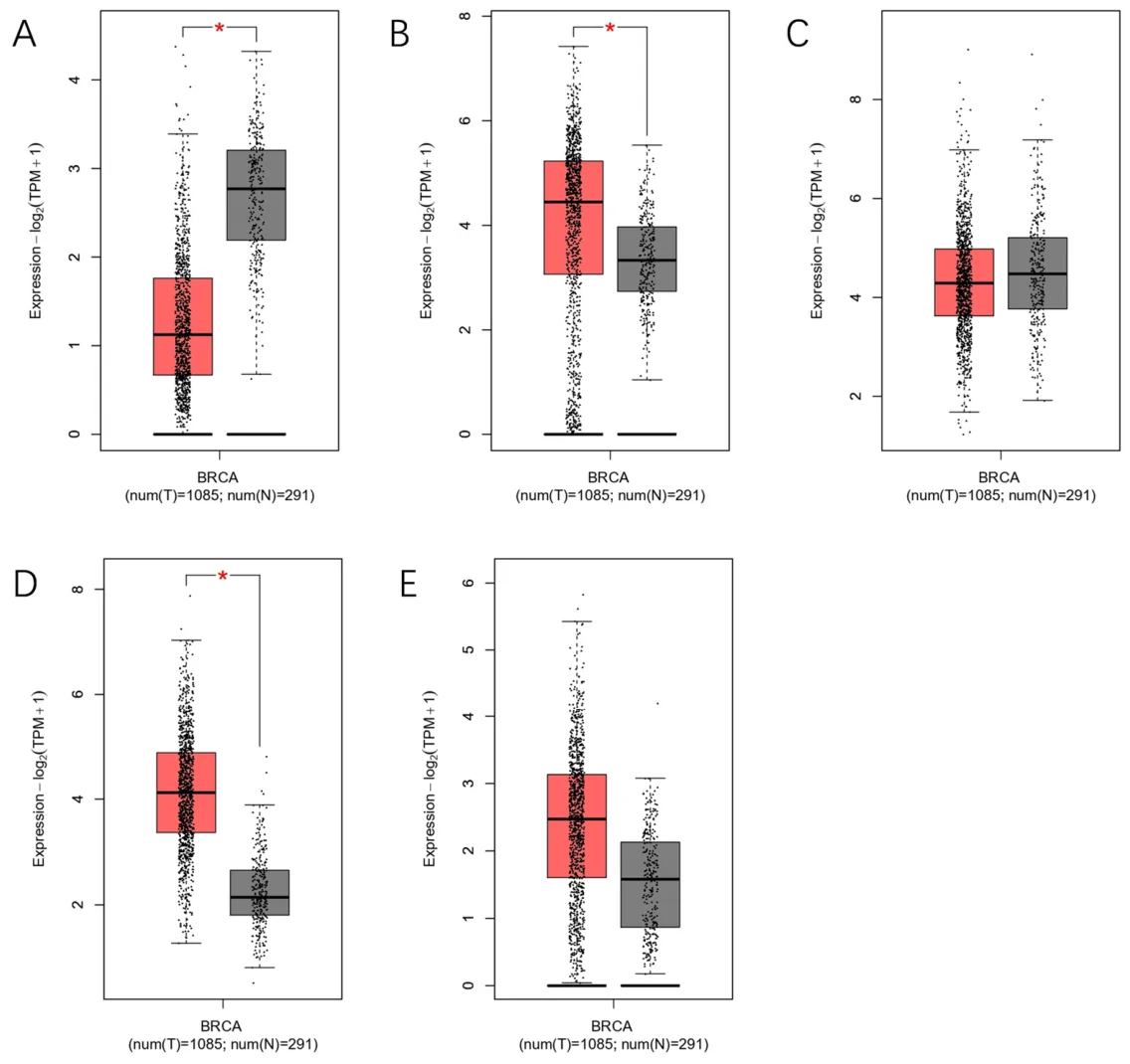
External validation of machine learning-identified core genes in the TCGA-BRCA cohort. The red boxes represent tumor tissues, and the gray boxes represent normal tissues. The asterisk indicates a statistically significant difference between the two groups. (**A**) NR3C2 expression between breast cancer and normal tissues. (**B**) AR expression between breast cancer and normal tissues. (**C**) TGM2 expression between breast cancer and normal tissues. (**D**) ATAD2 expression between breast cancer and normal tissues. (**E**) P2RY2 expression between breast cancer and normal tissues.

As shown in Figure 9, NR3C2 expression was significantly lower in breast cancer tissues than in normal breast tissues (*p* < 0.05) (Figure 9A), whereas AR and ATAD2 expression levels were significantly elevated in breast cancer tissues compared with normal tissues (both *p* < 0.05) (Figure 9B,D). Although TGM2 and P2RY2 exhibited differences in expression between tumor and normal tissues, these differences did not reach statistical significance (both *p* > 0.05) (Figure 9C,E).

Based on integrated analyses of the GEO training datasets, machine learning models, SHAP interpretation results, and the TCGA external validation cohort, NR3C2 demonstrated a consistent downregulation pattern across multiple datasets. Moreover, NR3C2 exhibited the highest individual diagnostic performance (AUC = 0.982) and showed a substantial contribution to model output. Therefore, NR3C2 was prioritized as the candidate gene for subsequent molecular docking analysis, molecular dynamics simulations, and in vitro experimental validation.

### 2.11. Molecular Docking Analysis of Lindane with Key Target Proteins

To further investigate the potential interactions between Lindane and the candidate target proteins, molecular docking analyses were performed using the CB-Dock2 platform(http://183.56.231.194:8001/cb-dock2/index.php, accessed on 10 May 2026).

Blind protein–ligand docking was performed separately for NR3C2, AR, TGM2, ATAD2, and P2RY2 proteins. The binding modes between Lindane and each target protein were subsequently analyzed based on the docked conformations with the lowest predicted binding energies.

**Figure 10:**
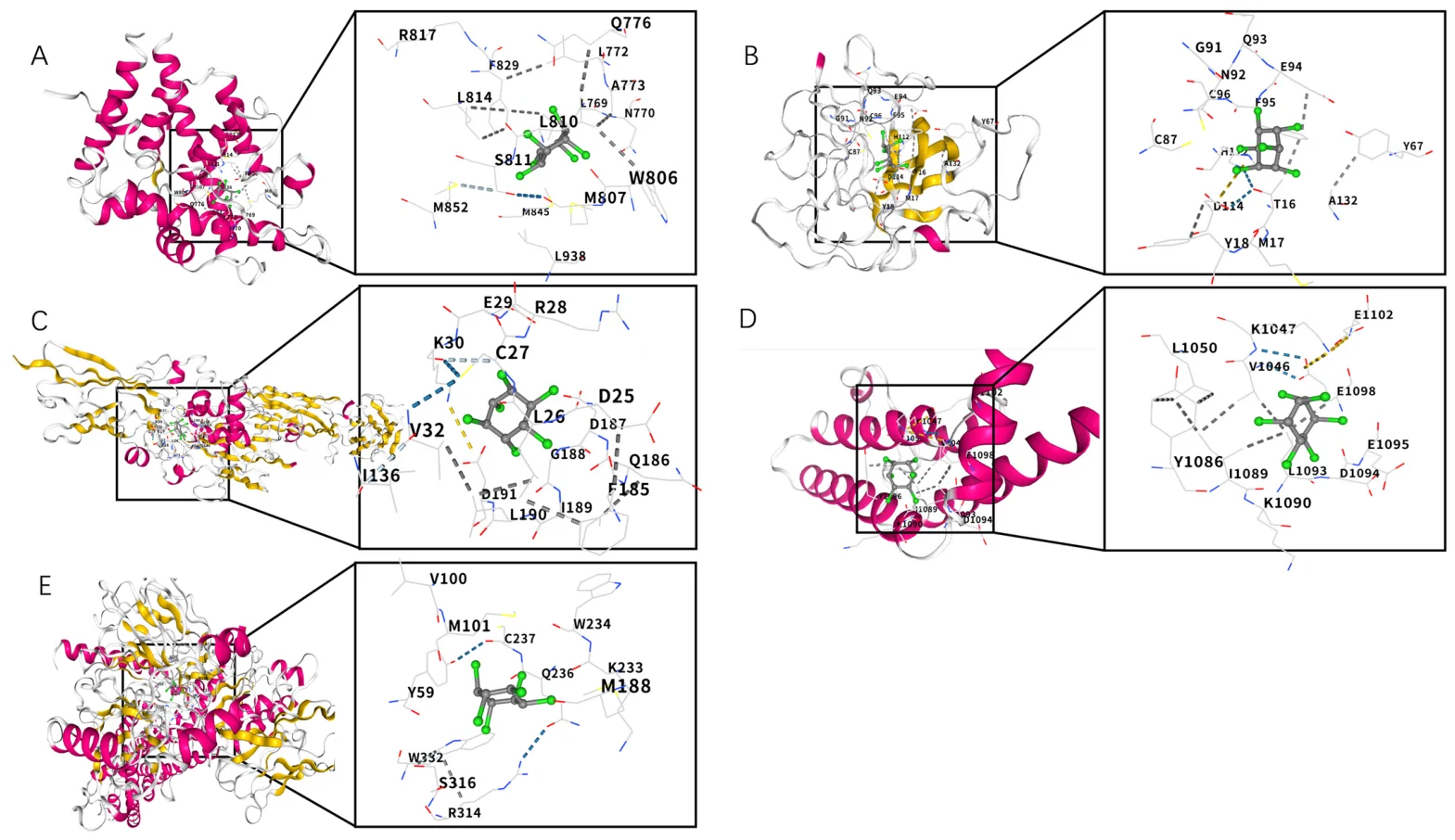
Molecular docking analysis between Lindane and candidate proteins. The dashed lines denote non-covalent interactions between ligand and protein. Blue and light blue dashed lines indicate hydrogen bonds and weak hydrogen bonds, respectively; gray dashed lines represent hydrophobic interactions; yellow dashed lines denote ionic interactions. (**A**) Predicted binding mode of Lindane with NR3C2 protein. (**B**) Predicted binding mode of Lindane with AR protein. (**C**) Predicted binding mode of Lindane with TGM2 protein. (**D**) Predicted binding mode of Lindane with ATAD2 protein. (**E**) Predicted binding mode of Lindane with P2RY2 protein.

The results showed that Lindane could enter the predicted binding pockets of the candidate proteins and adopt energetically favourable conformations with appropriate spatial compatibility (Figure 10). Notably, the NR3C2–Lindane and P2RY2–Lindane complexes exhibited relatively low predicted binding energies of −6.2 and −5.5 kcal/mol, respectively, suggesting potentially favourable interactions between Lindane and these proteins.

Further visualization and analysis of representative binding modes were performed using PyMOL2.6 and Discovery Studio4.5 software. The results demonstrated that the predicted interaction between Lindane and NR3C2 was mainly mediated by non-covalent interactions, including hydrophobic interactions and van der Waals forces.

These findings indicated a potential molecular interaction between Lindane and NR3C2, providing computational support for subsequent molecular dynamics simulations and experimental validation.

To further validate the reliability of the molecular docking results obtained from CB-Dock2, an independent docking analysis was performed using SwissDock (https://www.swissdock.ch/, accessed on 1 October 2026). The SwissDock-predicted docking scores showed comparable trends with those obtained from CB-Dock2 for the selected core targets, indicating consistent potential interactions between Lindane and these proteins. The detailed validation results, including the docking scores and predicted binding conformations obtained from SwissDock, are provided in Supplementary Table S1 and Supplementary Figure.

### 2.12. Molecular Dynamics Simulation Analysis of the NR3C2–Lindane Complex

To further evaluate the structural stability of the NR3C2–Lindane complex under dynamic conditions, a 100 ns molecular dynamics simulation was performed using the optimal docking conformation as the initial structure.

**Figure 11:**
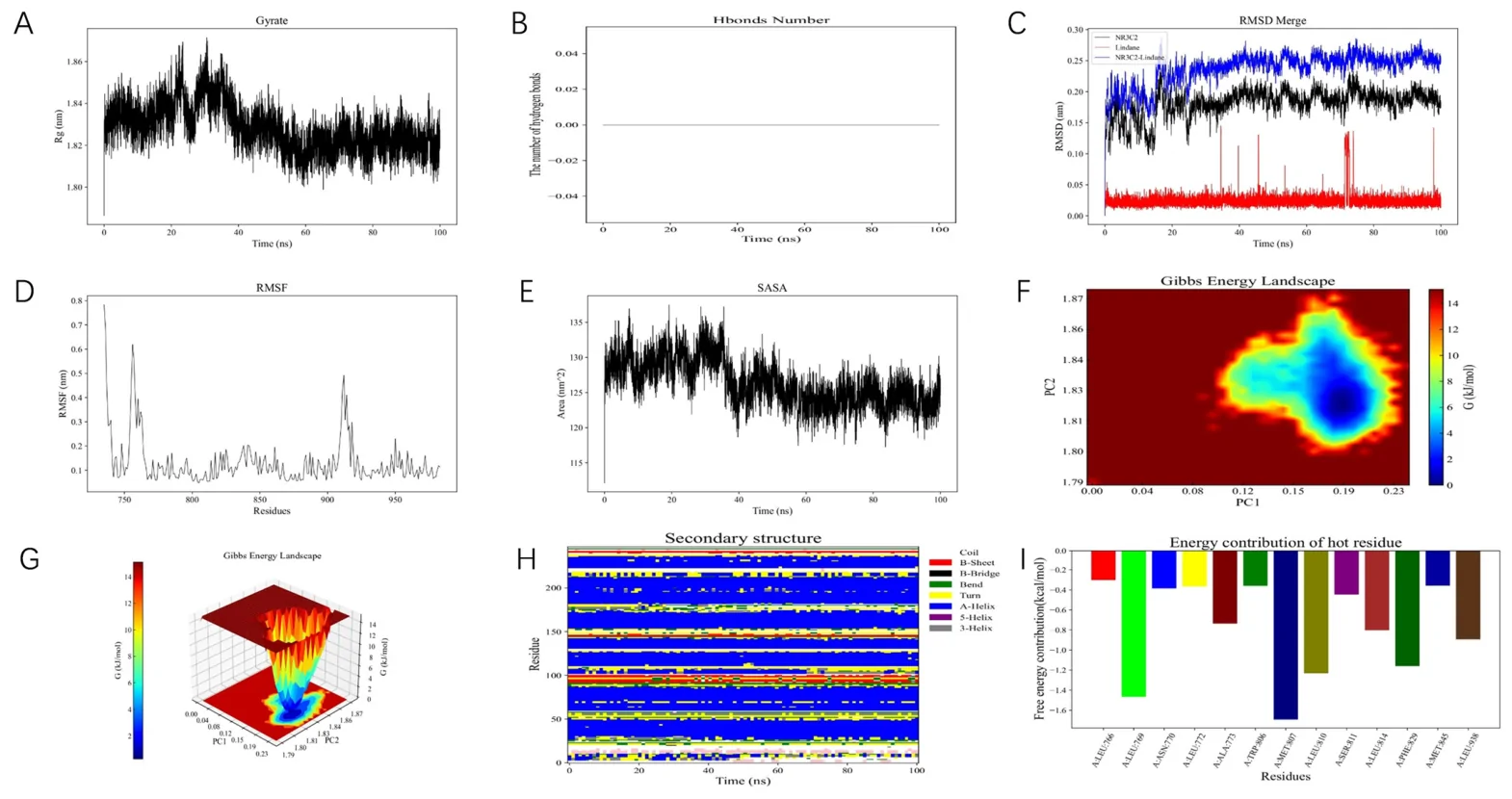
Molecular dynamics simulation of NR3C2–Lindane complex. (**A**) Radius of gyration (Rg) analysis during 100 ns simulation. (**B**) Hydrogen bond analysis between NR3C2 and Lindane. (**C**) RMSD analysis of protein–ligand complex, protein backbone and ligand. (**D**) RMSF analysis of NR3C2 residues. (**E**) SASA analysis during simulation. (**F**) MM/GBSA binding free energy decomposition. (**G**) 3D Gibbs Free Energy Landscape. (**H**) Secondary structure evolution heatmap over time. (**I**) Per-residue decomposition of the energy contribution.

The radius of gyration (Rg) was first analyzed to evaluate the compactness and global structural properties of the complex (Figure 11A). During the 100 ns simulation, the average Rg value of the NR3C2–Lindane complex was maintained at 1.83 ± 0.01 nm, with only minor fluctuations observed throughout the trajectory. These results suggested that the NR3C2–Lindane complex maintained an overall compact and equilibrated conformation during the simulation.

Hydrogen-bond analysis was subsequently performed to characterize ligand–protein interactions. The average number of hydrogen bonds between NR3C2 and Lindane during the simulation was 0 ± 0, suggesting that hydrogen bonding was unlikely to be a major contributor to complex formation. Instead, the complex was likely maintained mainly through non-hydrogen-bond interactions, including hydrophobic contacts and van der Waals forces (Figure 11B).

RMSD analysis showed that the average RMSD values of the NR3C2–Lindane complex, apo-NR3C2 protein, and Lindane molecule were 0.24 ± 0.02 nm, 0.18 ± 0.02 nm, and 0.02 ± 0.01 nm, respectively (Figure 11C). The relatively stable RMSD profiles indicated that the complex exhibited stable conformational behavior during the simulation period without substantial structural deviation.

RMSF analysis was performed to assess residue-level flexibility of NR3C2 during the simulation (Figure 11D). The average RMSF value of NR3C2 was 0.13 ± 0.10 nm, indicating limited residue fluctuations. Only several localized regions showed relatively higher flexibility, which may correspond to intrinsically flexible loop regions.

SASA analysis demonstrated that the average solvent-accessible surface area of NR3C2 was 126.40 ± 3.25 nm^2^, with minimal fluctuations throughout the simulation (Figure 11E). These results suggested that Lindane association was not accompanied by substantial changes in the solvent exposure of NR3C2.

The binding free energy of the NR3C2–Lindane complex was further calculated using the MM/GBSA approach (Figure 11F,G). The total binding free energy (ΔTotal) was −19.14 ± 2.13 kcal/mol. The van der Waals interaction energy (ΔVDWAALS) contributed −27.87 ± 0.78 kcal/mol, whereas the electrostatic interaction energy (ΔEelec) contributed −5.32 ± 1.47 kcal/mol. Although the solvation free energy (ΔGsolvation) showed an unfavorable contribution (14.06 ± 1.33 kcal/mol), the overall negative binding free energy suggested an energetically favorable interaction between NR3C2 and Lindane, predominantly driven by van der Waals and other non-polar interactions.

Secondary structure analysis revealed that the major structural elements of NR3C2, including α-helices, β-sheets, and turns, remained largely unchanged throughout the 100 ns simulation (Figure 11H). These findings indicated that Lindane association was not accompanied by substantial secondary structural rearrangement.

Per-residue energy decomposition further identified several amino acid residues that contributed substantially to the binding free energy of the NR3C2–Lindane complex (Figure 11I). These residues may participate in ligand recognition through hydrophobic and van der Waals interactions.

Collectively, the molecular dynamics simulation results demonstrated that the NR3C2–Lindane complex maintained a compact and equilibrated conformation during the simulation period. The stable RMSD, RMSF, Rg, and SASA profiles, together with the favorable MM/GBSA binding energy, provided computational evidence supporting a potential interaction between Lindane and NR3C2.

### 2.13. Lindane Reduces the Viability of Cells

To validate the bioinformatics predictions, in vitro cellular experiments were further conducted to investigate the effects of Lindane on cell viability. First, a CCK-8 assay was performed to evaluate changes in cell viability following treatment with different concentrations of Lindane.

**Figure 12:**
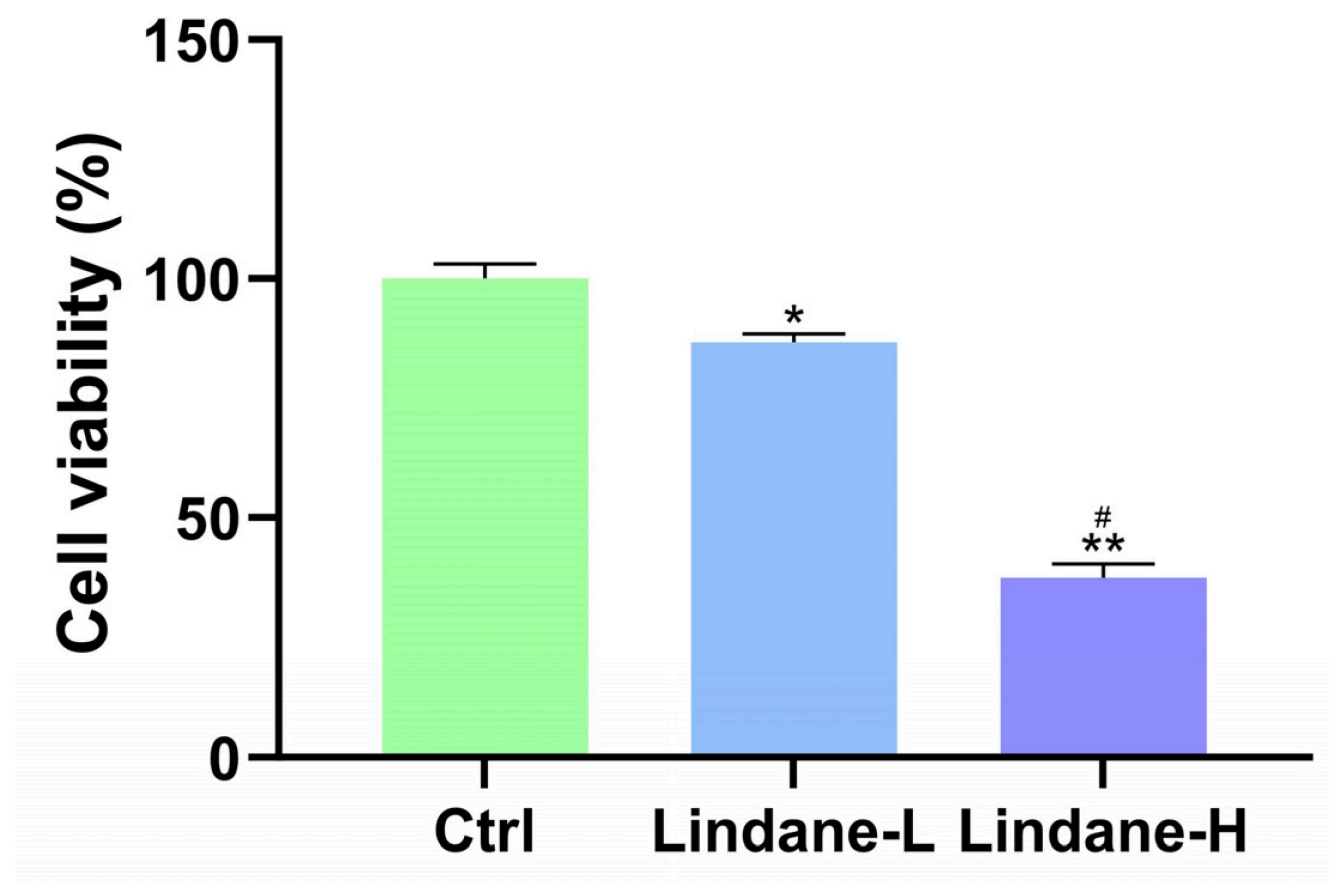
Effect of Lindane on cell viability. Cell viability was measured using the CCK-8 assay after treatment with low- or high-dose Lindane. Data are presented as mean ± SD from *n* = 3 independent experiments. Statistical significance was analyzed using one-way ANOVA followed by Dunnett’s multiple comparisons test. * *p* < 0.05 and ** *p* < 0.01 versus the Ctrl group; # *p* < 0.05 versus the Lindane-L group.

The results showed that Lindane treatment significantly decreased cell viability in a dose-dependent manner. As the concentration of Lindane increased, cell viability progressively declined, with a significant reduction observed at 10 μM and a stronger inhibitory effect observed at 20 μM. Therefore, 10 μM and 20 μM Lindane were selected as the low- and high-dose treatment concentrations (Lindane-L and Lindane-H, respectively) for subsequent experiments. Statistical analysis revealed significant differences among the treatment groups (*p* < 0.01 compared with the Ctrl group; *p* < 0.05 compared with the Lindane-L group) (Figure 12).

### 2.14. Lindane Induces Oxidative Stress Responses in Cells

**Figure 13:**
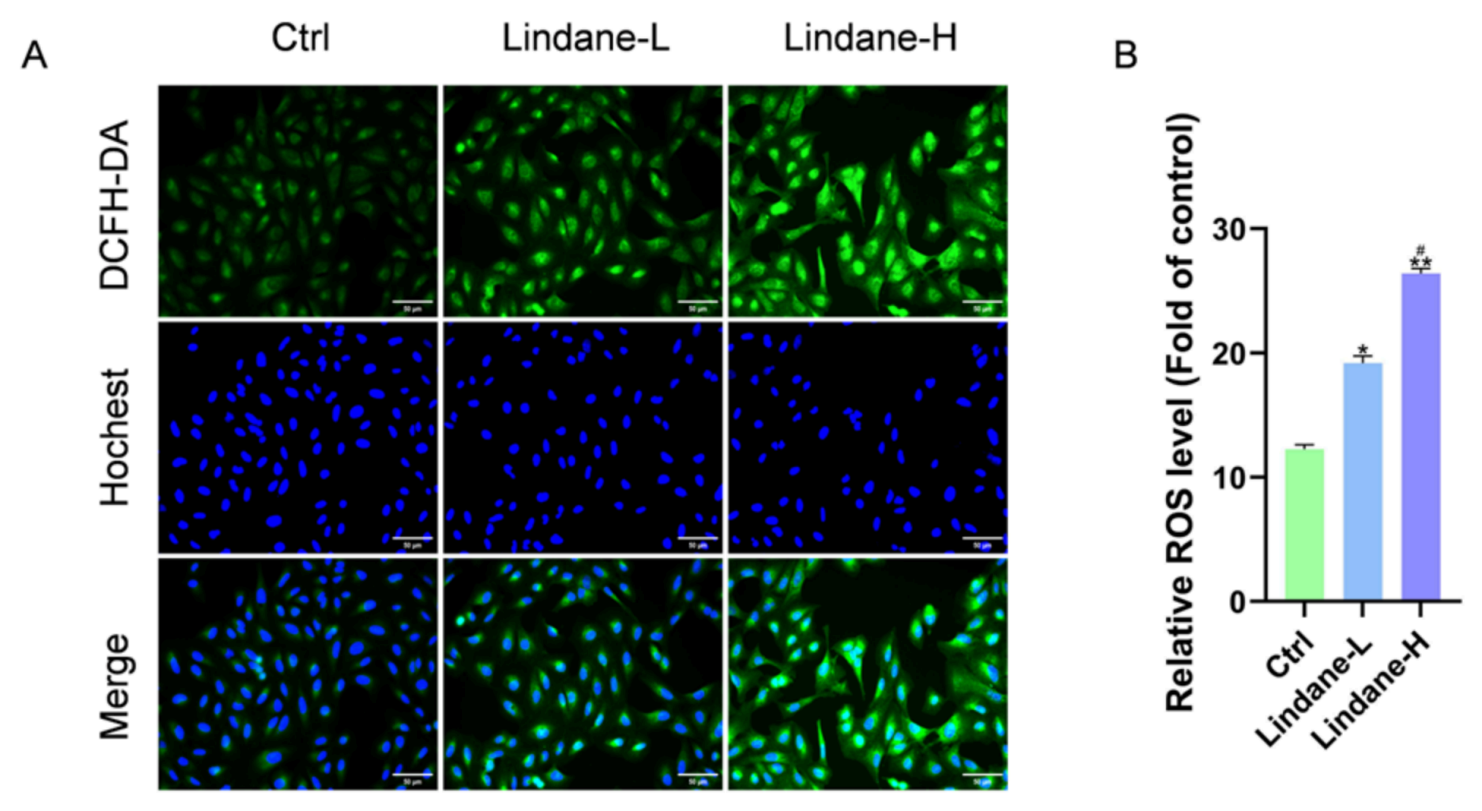
Effect of Lindane on intracellular ROS accumulation. (**A**) Intracellular ROS levels were measured using the DCFH-DA fluorescent probe after Lindane treatment. The green fluorescence indicates DCFH-DA staining for ROS, and blue fluorescence represents Hoechst staining for cell nuclei. (**B**) Data are presented as mean ± SD from *n* = 3 independent experiments. Statistical significance was analyzed using one-way ANOVA followed by Tukey’s multiple comparisons test. * *p* < 0.05 and ** *p* < 0.01 versus the Ctrl group; # *p* < 0.05 versus the Lindane-L group.

To investigate whether Lindane induces oxidative stress in cells, intracellular ROS levels were measured using DCFH-DA fluorescence analysis. The relative ROS levels in the Ctrl, Lindane-L, and Lindane-H groups were 12.26 ± 0.34, 19.16 ± 0.58, and 26.39 ± 0.40, respectively (Figure 13A). The results demonstrated that Lindane treatment significantly increased intracellular ROS accumulation in cells. Compared with the Ctrl group, ROS levels increased by approximately 56.3% and 115.2% in the Lindane-L and Lindane-H groups, respectively. Moreover, the Lindane-H group exhibited significantly higher ROS accumulation than the Lindane-L group. One-way ANOVA followed by post hoc multiple comparison analysis revealed significant differences among the treatment groups (*p* < 0.01 vs. Ctrl group; *p* < 0.05 vs. Lindane-L group) (Figure 13B). Collectively, these findings indicate that Lindane promotes intracellular ROS accumulation in cells in a dose-dependent manner.

### 2.15. Lindane Promotes Apoptosis in Cells

**Figure 14:**
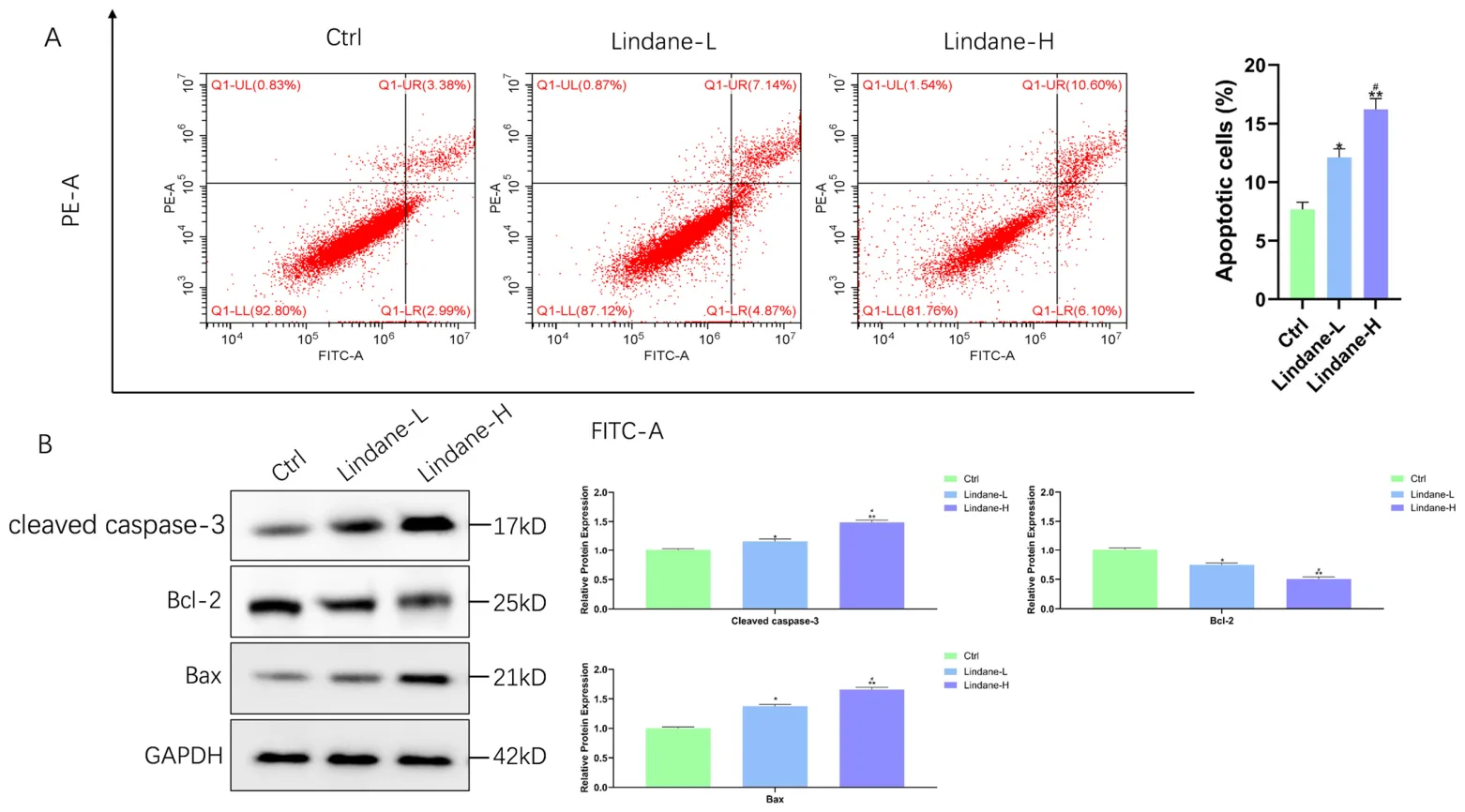
Lindane promotes apoptosis and alters apoptosis-related protein expression in cells. (**A**) Representative flow cytometric plots of Annexin V-FITC/PI-stained cells in the Ctrl, Lindane-L, and Lindane-H groups. The lower-left quadrant represents viable cells, the lower-right quadrant represents early apoptotic cells, the upper-right quadrant represents late apoptotic or secondary necrotic cells, and the upper-left quadrant represents necrotic cells. The total apoptotic rate was calculated as the sum of early and late apoptotic cells. The bar graph shows the quantitative analysis of total apoptotic cells. (**B**) Data are presented as mean ± SD from *n* = 3 independent experiments. Statistical significance was analyzed using one-way ANOVA followed by Tukey’s multiple comparisons test. * *p* < 0.05 and ** *p* < 0.01 versus the Ctrl group; # *p* < 0.05 versus the Lindane-L group.

Annexin V-FITC/PI double staining combined with flow cytometry was performed to evaluate apoptosis levels in cells following Lindane treatment. Representative flow cytometry profiles demonstrated that Lindane exposure increased the proportions of both early and late apoptotic cells. The total apoptosis rate was calculated by summing the percentages of early and late apoptotic cells. The apoptosis rates in the Ctrl, Lindane-L, and Lindane-H groups were 6.37%, 12.01%, and 16.70%, respectively. Quantitative analysis further revealed that Lindane treatment significantly increased the overall apoptosis rate compared with the Ctrl group, with a more pronounced effect observed in the Lindane-H group. Moreover, the apoptosis rate in the Lindane-H group was significantly higher than that in the Lindane-L group, indicating that Lindane induces apoptosis in cells in a dose-dependent manner (Figure 14A).

Western blot analysis further supported the flow cytometry results. Following Lindane treatment, the expression levels of the pro-apoptotic proteins Bax and cleaved caspase-3 were significantly increased, whereas the expression level of the anti-apoptotic protein Bcl-2 was decreased. These alterations were more pronounced in the Lindane-H group compared with the Lindane-L group. Collectively, these findings demonstrate that Lindane treatment induces molecular alterations associated with apoptosis in cells, characterized by increased expression of pro-apoptotic proteins and reduced expression of anti-apoptotic proteins (Figure 14B).

### 2.16. Lindane Regulates the Expression of NR3C2, TGM2, and ATAD2

**Figure 15:**
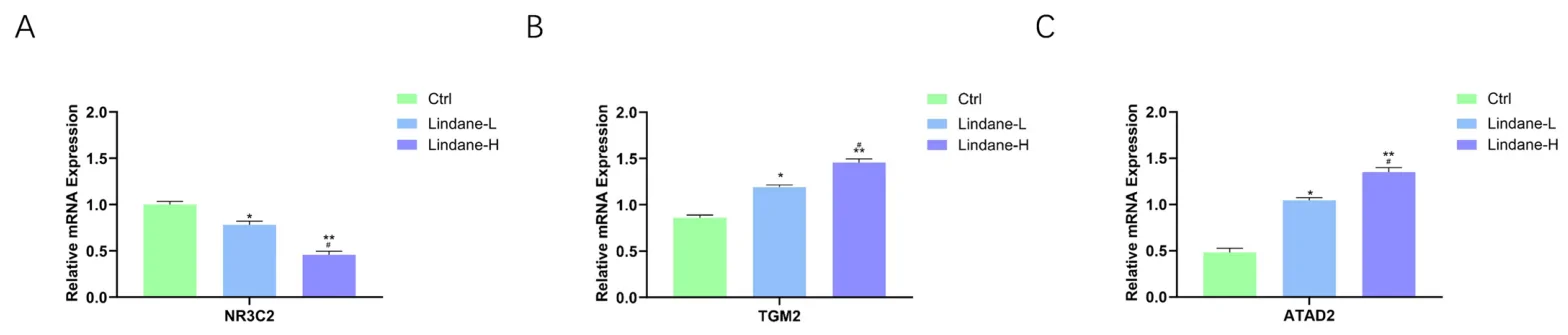
Effects of Lindane on the mRNA expression of NR3C2, TGM2, and ATAD2. (**A**) Relative mRNA expression level of NR3C2 determined by qPCR. (**B**) Relative mRNA expression level of TGM2 determined by qPCR. (**C**) Relative mRNA expression level of ATAD2 determined by qPCR. The relative mRNA expression levels were determined by quantitative real-time PCR and calculated using the 2^−ΔΔCt^ method, with GAPDH used as the internal reference. Data are presented as mean ± SD from *n* = 3 independent experiments. Statistical significance was analyzed using one-way ANOVA followed by Tukey’s multiple comparisons test. * *p* < 0.05 and ** *p* < 0.01 versus the Ctrl group; # *p* < 0.05 versus the Lindane-L group.

qRT-PCR analysis demonstrated that Lindane treatment significantly altered the mRNA expression levels of the core candidate genes. Compared with the Ctrl group, NR3C2 mRNA expression levels decreased to 0.782 ± 0.040 and 0.458 ± 0.038 in the Lindane-L and Lindane-H groups, respectively. Notably, the Lindane-H group exhibited a further decrease in NR3C2 expression compared with the Lindane-L group, indicating a dose-dependent downregulation pattern. In contrast, TGM2 mRNA expression increased to 1.385 ± 0.029 and 1.698 ± 0.045, whereas ATAD2 mRNA expression increased to 2.169 ± 0.061 and 2.804 ± 0.099 in the Lindane-L and Lindane-H groups, respectively. One-way ANOVA followed by post hoc multiple comparison analysis revealed significant differences among the treatment groups (*p* < 0.01 vs. Ctrl group; *p* < 0.05 vs. Lindane-L group) (Figure 15). These findings indicate that Lindane exposure is associated with transcriptional suppression of NR3C2 and upregulation of TGM2 and ATAD2, with ATAD2 showing the greatest magnitude of upregulation. Collectively, these results suggest that Lindane exposure may contribute to Lindane-induced cellular responses in cells through modulation of candidate gene expression.

### 2.17. Lindane Regulates the Expression of Apoptosis-Related Proteins

**Figure 16:**
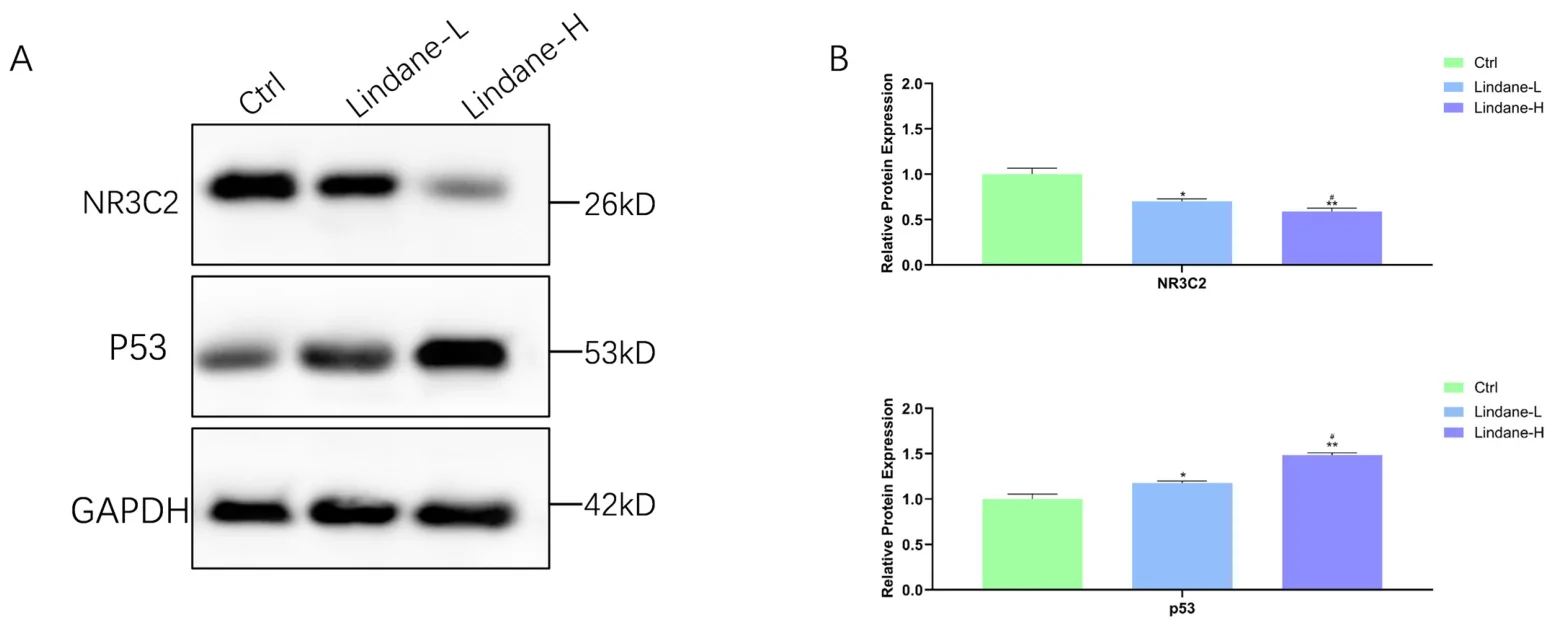
Effects of Lindane on NR3C2 and p53 protein expression in cells. (**A**) Representative Western blot bands showing NR3C2 and p53 protein expression in the Ctrl, Lindane-L, and Lindane-H groups. GAPDH was used as the loading control. (**B**) Densitometric quantification of NR3C2 and p53 expression normalized to GAPDH. Data are presented as mean ± SD from *n* = 3 independent experiments. Statistical significance was analyzed using one-way analysis of variance (ANOVA) followed by Tukey’s multiple comparisons test. * *p* < 0.05 and ** *p* < 0.01 versus the Ctrl group; # *p* < 0.05 versus the Lindane-L group.

Western blot analysis further demonstrated that Lindane treatment decreased NR3C2 protein expression while increasing p53 protein expression, with these alterations being more evident in the Lindane-H group. Densitometric quantification confirmed that, compared with the Ctrl group, Lindane exposure significantly reduced NR3C2 protein levels and elevated p53 protein levels (*p* < 0.01 vs. Ctrl group; *p* < 0.05 vs. Lindane-L group) (Figure 16).

These findings suggest that Lindane-induced apoptosis is associated with reduced NR3C2 expression and increased p53 expression, which may contribute to the activation of apoptosis-related responses.

### 2.18. Comprehensive Analysis of Mechanisms of Action

By integrating the results of network toxicology analysis, machine learning screening, molecular modelling, and cellular experiments, this study provides insights into the potential mechanism underlying Lindane-associated biological effects in breast cancer.

The results suggest that Lindane exposure may be involved in the regulation of lipid metabolism-related processes, oxidative stress responses, and apoptosis through multiple molecular networks. Among these candidates, NR3C2, identified as a key candidate gene through machine learning analysis, showed consistent evidence across expression validation, SHAP contribution analysis, and molecular modelling. In vitro experiments further demonstrated that Lindane treatment was associated with decreased NR3C2 expression, increased ROS accumulation, and alterations in apoptosis-related proteins.

Therefore, NR3C2 may represent an important candidate molecular node within the regulatory network underlying Lindane-associated biological effects in breast cancer.

## 3. Discussion

Breast cancer development and progression involve complex interactions among genetic alterations, hormonal regulation, metabolic reprogramming, oxidative stress, and the tumor immune microenvironment. Increasing evidence suggests that persistent organic pollutants (POPs) with endocrine-disrupting properties and bioaccumulative characteristics may contribute to hormone-related cancer development. Lindane, a representative organochlorine pesticide, exhibits strong lipophilicity, environmental persistence, and long-term accumulation potential in biological tissues. However, the molecular mechanisms underlying Lindane-associated breast cancer-related alterations remain poorly understood. In this study, we integrated network toxicology, bioinformatics, machine learning, molecular modelling, and in vitro experiments to identify and validate potential molecular candidates involved in Lindane-associated breast cancer responses. Our findings highlight NR3C2 as a potential molecular node associated with Lindane exposure and breast cancer-related biological alterations.

Integrated target prediction and disease association analyses identified overlapping molecular targets between Lindane exposure and breast cancer. Functional enrichment analysis suggested that these targets were mainly involved in lipid metabolism-related processes, mitochondrial functions, organic compound transport, and molecular binding activities. These findings are biologically plausible considering the high lipophilicity of Lindane and its potential influence on cellular metabolic processes. In addition, KEGG pathway analysis indicated enrichment of sphingolipid metabolism and p53-related pathways, suggesting that Lindane-associated molecular alterations may involve metabolic stress responses and apoptosis-related processes.

To prioritize biologically relevant candidates, we established a machine learning-based screening framework using multiple algorithm combinations and independent GEO datasets. The LASSO-plsRglm model exhibited strong predictive performance and identified five candidate hub genes, including NR3C2, AR, TGM2, ATAD2, and P2RY2. Among these candidates, NR3C2 showed the highest individual diagnostic performance and the strongest contribution according to SHAP analysis. Unlike conventional machine learning approaches that lack interpretability, SHAP analysis enabled quantitative evaluation of the contribution and direction of individual genes, improving the transparency, reproducibility, and biological interpretability of model-based candidate selection.

Validation using the TCGA-BRCA dataset further supported the relevance of these candidate genes in breast cancer. NR3C2 expression was consistently reduced in tumor tissues, whereas AR and ATAD2 exhibited increased expression patterns. NR3C2 encodes the mineralocorticoid receptor (MR), a member of the nuclear receptor superfamily involved in hormone regulation, metabolic processes, and cellular stress responses. Increasing evidence indicates that NR3C2 participates in cancer-related biological processes through transcriptional regulation and interaction with downstream signaling pathways. Previous studies have demonstrated that NR3C2 dysregulation is associated with breast cancer progression. In breast cancer cell models, NR3C2 restoration was reported to suppress cell proliferation, migration, and invasion, suggesting a potential tumor-regulatory role of NR3C2. Furthermore, recent studies have shown that NR3C2 contributes to triple-negative breast cancer growth through the PI3K/AKT pathway. Other studies have demonstrated that MR/NR3C2 signaling regulates aggressive tumor phenotypes, including migration and metastatic potential [20,21,22].

Immune infiltration analysis suggested that candidate genes were associated with distinct immune cell populations, including macrophages, mast cells, and memory T cells. These findings indicate that Lindane-associated molecular alterations may be linked with immune-related characteristics within the tumor microenvironment. Given the important roles of these immune populations in inflammation, immune surveillance, and tissue remodeling, alterations in candidate gene expression may be associated with variations in immune-associated biological states. However, further experimental studies are required to clarify the specific relationships among Lindane exposure, candidate genes, and immune regulation.

Single-gene GSVA revealed that candidate genes were associated with diverse biological processes, including glucose metabolism, protein degradation, DNA repair, cell cycle regulation, lipid metabolism, and immune-related signaling. Among these genes, NR3C2 showed potential associations with metabolic regulation and stress-response pathways, further supporting its potential importance within the molecular network influenced by Lindane exposure.

Molecular docking and molecular dynamics simulations provided structural evidence supporting a potential interaction between Lindane and candidate proteins. Among the identified candidate proteins, NR3C2 exhibited favorable binding characteristics with Lindane. The NR3C2–Lindane complex demonstrated stable conformational behavior during a 100 ns molecular dynamics simulation, with consistent RMSD, RMSF, Rg, and SASA profiles. MM/GBSA analysis suggested that the interaction was mainly stabilized by van der Waals and hydrophobic interactions. Although persistent hydrogen bonds were not observed during simulation, these computational results provide additional evidence supporting NR3C2 as a potential molecular target associated with Lindane exposure. When interpreted together with the subsequent in vitro findings, these computational results provide complementary evidence supporting the potential involvement of NR3C2 in the cellular response to Lindane exposure.

In the present study, MCF-7 breast cancer cells were used to evaluate the cellular responses to Lindane exposure. Lindane treatment significantly reduced cell viability, increased intracellular ROS accumulation, promoted apoptosis, upregulated p53, Bax, and cleaved caspase-3 expression, and downregulated Bcl-2 and NR3C2 expression. These findings suggest that Lindane exposure is associated with oxidative stress-associated apoptotic responses in MCF-7 cells and support the molecular alterations predicted by the network toxicology analysis. Because breast cancer is a heterogeneous disease, the present findings should be interpreted within the context of the MCF-7 cell model. Other breast cancer cell lines, such as MDA-MB-231, possess distinct molecular characteristics and apoptosis-related signaling pathways, which may lead to different responses to Lindane exposure. Therefore, the molecular responses to Lindane may differ among different breast cancer cell subtypes. Additional studies using MDA-MB-231 cells are warranted to determine whether the molecular alterations observed in MCF-7 cells are conserved across different breast cancer subtypes. Similar observations have been reported in previous studies showing that organochlorine pesticides, including Lindane, are associated with oxidative stress, endocrine disruption, and apoptosis-related molecular alterations in breast cancer models [23]. These published findings are generally consistent with our experimental observations.

Consistent with the cellular findings, qRT-PCR and Western blot analyses showed a dose-dependent reduction in NR3C2 expression following Lindane treatment, accompanied by increased p53, Bax, and cleaved caspase-3 expression and decreased Bcl-2 expression. These findings indicate that Lindane exposure alters NR3C2 expression concurrently with apoptosis-related molecular responses. Although the present study does not determine whether NR3C2 directly mediates Lindane-induced apoptosis, the observed changes in NR3C2 expression, together with apoptosis-associated molecular alterations, suggest that NR3C2 may be involved in the cellular response to Lindane exposure. Therefore, the relationship between NR3C2 dysregulation and apoptosis should be interpreted as an association rather than a causal mechanism. Further studies involving NR3C2 knockdown, overexpression, or rescue experiments are required to clarify its precise functional role.

Collectively, this study proposes a potential regulatory model in which Lindane exposure may contribute to breast cancer-associated biological alterations through alterations potentially involving metabolic processes, oxidative stress responses, apoptosis-associated processes, and NR3C2-related molecular changes. The integration of computational prediction and experimental validation provides a comprehensive framework for investigating environmental pollutant-associated cancer mechanisms.

Several limitations should be acknowledged. First, although multiple public datasets were integrated, differences in sequencing platforms, sample characteristics, and clinical backgrounds may influence gene expression patterns. Second, machine learning models require further validation in larger and more diverse cohorts. Finally, functional experiments involving NR3C2 knockdown, overexpression, and rescue approaches are required to clarify the precise mechanistic role of NR3C2 in Lindane-associated breast cancer responses.

## 4. Materials and Methods

### 4.1. Identification of Potential Lindane Targets

The chemical information and predicted targets of Lindane were obtained from publicly available databases. The PubChem database was searched using “Lindane” as the keyword to obtain its canonical chemical structure and SMILES notation [24]. The retrieved SMILES string was subsequently submitted to the ChEMBL (https://www.ebi.ac.uk/chembl/, accessed on 20 January 2026), STITCH (https://ngdc.cncb.ac.cn/databasecommons/database/id/208, accessed on 20 January 2026), and SuperPred (https://bio.tools/superpred, accessed on 20 January 2026) databases for target prediction. Additionally, the SMILES string was uploaded to the Swiss Target Prediction platform (http://www.swisstargetprediction.ch/, accessed on 20 January 2026), and targets with a non-zero prediction probability were retained [24]. The predicted targets from all databases were integrated, redundant entries were removed, and target information was standardized using the UniProt database (https://www.uniprot.org/, accessed on 26 January 2026). Finally, a comprehensive Lindane target library was established for subsequent bioinformatics and network toxicology analyses [25].

### 4.2. Acquisition and Preprocessing of Breast Cancer-Related Transcriptomic Data

The human breast cancer-related gene expression datasets were retrieved from the Gene Expression Omnibus (GEO) database. The keywords “Breast cancer” and “Homo sapiens” were used for dataset retrieval, and five datasets meeting the inclusion criteria were selected, including GSE22820, GSE26304, GSE57297, GSE139038, and GSE205185.

The GSE22820 and GSE26304 datasets were merged to establish the training cohort and were used for differential expression analysis, weighted gene co-expression network analysis (WGCNA), and machine learning model construction. The GSE57297, GSE139038, and GSE205185 datasets were employed as independent external validation cohorts to assess the robustness and generalizability of the identified biomarkers and predictive models.

To reduce batch effects arising from differences in experimental platforms and processing procedures, the GSE22820 and GSE26304 datasets were integrated, and batch correction was performed using the “SVA” package in R4.3.3 software [26]. The resulting batch-corrected expression matrix was subsequently used for downstream bioinformatics analyses. Detailed information regarding the included datasets is summarized in Table 1.

### 4.3. Differential Expression Analysis

The integrated GEO expression matrix was analyzed using the “limma” package in R software (version 4.4.1) to identify differentially expressed genes (DEGs) [27]. The gene expression profiles between normal control samples and breast cancer (BC) samples were compared to screen BC-associated DEGs. The linear model implemented in the limma package was used to estimate differential expression, and the Benjamini–Hochberg method was applied for multiple testing correction. DEGs were screened according to the criteria of |log_2_FC| > 0.585 and adjusted *p*-value < 0.05. Volcano plots were generated using the “ggplot2” package to visualize the distribution of DEGs, and heatmaps were constructed to illustrate the expression patterns of the identified DEGs.

### 4.4. Weighted Gene Co-Expression Network Analysis (WGCNA)

To identify gene modules associated with breast cancer (BC) status, weighted gene co-expression network analysis (WGCNA) was performed using the “WGCNA” package in R software [28]. The gene expression matrix was first subjected to sample quality assessment, and network topology parameters were evaluated under different soft-threshold powers. The optimal soft-threshold power was selected according to the scale-free network topology criterion. An adjacency matrix was constructed using the optimal soft-threshold power and subsequently transformed into a TOM to minimize the influence of spurious correlations among genes.

Hierarchical clustering was then performed based on the TOM, and genes with similar expression patterns were assigned to distinct modules. Module eigengenes were calculated, and module–trait relationship analysis was conducted to assess the correlations between module eigengenes and BC status. Modules significantly associated with BC status were identified as key modules, and genes contained within these modules were extracted for subsequent analyses.

### 4.5. Identification of Lindane–Breast Cancer Common Targets

To identify candidate targets involved in Lindane-associated breast cancer toxicity, breast cancer-related genes were integrated with predicted Lindane targets for further screening. The DEGs obtained from differential expression analysis were intersected with key module genes identified by WGCNA, and redundant genes were removed to generate a breast cancer-related gene set. Subsequently, this gene set was intersected with predicted Lindane targets to identify overlapping targets. These overlapping targets were defined as Lindane–breast cancer candidate targets and were further subjected to functional enrichment analysis, machine learning-based feature selection, and mechanistic investigation.

### 4.6. GO and KEGG Enrichment Analysis

To investigate the potential biological functions and signaling pathways associated with Lindane–breast cancer candidate targets, Gene Ontology (GO) and Kyoto Encyclopedia of Genes and Genomes (KEGG) enrichment analyses were performed using the “cluster Profiler” package in R software. GO analysis was conducted based on three functional categories, including Biological Process (BP), Cellular Component (CC), and Molecular Function (MF), to characterize the biological roles of the candidate targets. KEGG enrichment analysis was further performed to identify signaling pathways associated with these targets. An adjusted *p*-value < 0.05 was considered statistically significant, and the enrichment results were visualized using R-based methods, including bubble plots, bar plots, and circular enrichment plots [29,30].

### 4.7. Machine Learning Model Construction and Core Gene Selection

To identify key genes with potential diagnostic value and develop a breast cancer prediction model, multiple machine learning algorithms were applied for feature selection and model construction. Twelve machine learning algorithms were included, namely LASSO regression, Ridge regression, stepwise generalized linear model (Stepglm), extreme gradient boosting (XGBoost), random forest (RF), elastic network, partial least squares generalized linear model (plsRglm), generalized boosted regression model (GBM), naive Bayes, linear discriminant analysis (LDA), glmBoost, and support vector machine (SVM) [31,32].

The models were constructed using the training cohort with a 10-fold cross-validation strategy, and 113 candidate models were generated by integrating different machine learning algorithms. The GSE57297, GSE139038, and GSE205185 datasets were subsequently used as independent external validation cohorts to evaluate model performance. The area under the receiver operating characteristic curve (AUC) was used as the primary evaluation metric, and the model achieving the highest average AUC across the validation cohorts was selected as the final predictive model [33].

### 4.8. SHAP Model Interpretation Analysis

To further improve the interpretability of the machine learning model, SHAP (Shapley Additive exPlanations) analysis was performed to quantify the contribution of each feature to model predictions. The SHAP summary plot was used to evaluate the overall importance of the identified biomarkers, and the mean absolute SHAP values were calculated to rank feature importance. Furthermore, SHAP dependence plots were generated to illustrate the relationship between gene expression levels and model predictions, whereas SHAP waterfall plots and force plots were used to visualize the contribution of individual features to individual sample predictions [34].

### 4.9. Immune Infiltration Analysis

To investigate the potential association between key genes and the breast cancer immune microenvironment, immune cell infiltration analysis was performed using the CIBERSORT algorithm in the training cohort. The relative proportions of 22 immune cell subsets were estimated based on the LM22 immune cell signature matrix. Spearman correlation analysis was conducted to evaluate the correlations between key gene expression levels and immune cell infiltration proportions. Furthermore, differences in immune cell compositions between breast cancer (BC) samples and normal control samples were compared, and the results were visualized using heatmaps and box plots [35].

### 4.10. Single-Gene GSVA

To further explore pathway alterations associated with key gene expression, single-gene pathway enrichment analysis was performed using gene set variation analysis (GSVA). Samples were divided into high-expression and low-expression groups based on key gene expression levels, and pathway enrichment scores were calculated using KEGG gene sets obtained from the MSigDB database. Differentially enriched pathways between the two groups were identified through statistical analysis, with an adjusted *p*-value < 0.05 considered statistically significant. The significantly enriched pathways were subsequently visualized [36].

### 4.11. Validation Analysis Using the TCGA Database

To further validate the expression patterns of key genes in breast cancer, the TCGA-BRCA dataset from The Cancer Genome Atlas (TCGA) database was used for independent expression validation [37]. The expression levels of key genes were compared between normal breast tissues and breast cancer tissues to determine whether their expression patterns were consistent with the GEO analysis results. The concordance of gene expression trends between GEO datasets and the TCGA-BRCA cohort was further evaluated to assess the reliability of the identified key genes.

### 4.12. Molecular Docking

To investigate the potential binding patterns and interaction characteristics between Lindane and candidate target proteins, molecular docking analysis was performed using the CB-Dock2 platform (http://183.56.231.194:8001/cb-dock2/index.php, accessed on 10 May 2026) [38]. CB-Dock2 is a protein–ligand blind docking tool based on protein cavity detection, which enables the prediction of potential ligand-binding sites, binding conformations, and docking scores for target proteins.

The three-dimensional structure file of Lindane was obtained from the PubChem database in SDF format. The SDF file was converted into mol2 format using Open Babel software2.4.1 and used as the ligand input file for molecular docking [39]. The three-dimensional crystal structures of candidate target proteins were retrieved from the RCSB Protein Data Bank (PDB) database [40]. PyMOL2.6 software was used for structural preprocessing, including the removal of crystallographic water molecules, co-crystallized ligands, and other unnecessary small molecules. The processed protein structures were subsequently saved in PDB format [41].

The processed candidate target protein structures and Lindane ligand structure were uploaded to the CB-Dock2 platform for blind molecular docking. The potential interactions between Lindane and candidate target proteins were evaluated based on docking scores, predicted binding pockets, and optimal binding conformations.

### 4.13. Molecular Dynamics Simulation

To further evaluate the structural stability and binding behavior of the NR3C2–Lindane complex, molecular dynamics (MD) simulation was performed. The optimal binding pose obtained from molecular docking was used as the initial conformation, and a 100 ns MD simulation was conducted for the complex system [42].

The structural stability and conformational dynamics of the NR3C2–Lindane complex were evaluated by analyzing structural changes and protein–ligand interaction patterns throughout the simulation. The main evaluation parameters included the root mean square deviation (RMSD) of the protein backbone, root mean square fluctuation (RMSF) of amino acid residues, radius of gyration (Rg), solvent-accessible surface area (SASA), and number of hydrogen bonds. Additionally, the molecular mechanics/generalized Born surface area (MM/GBSA) method was applied to estimate the binding free energy and further characterize the interaction stability of the NR3C2–Lindane complex.

### 4.14. Cell Culture Reagents and Experimental Equipment

Human MCF-7 breast cancer cells were purchased from Procell Life Science & Technology Co., Ltd. (Wuhan, China; Cat. No. CL-0149) [43]. Cells were cultured in high-glucose DMEM supplemented with 10% fetal bovine serum (FBS), 1% penicillin–streptomycin, and 0.01 mg/mL recombinant human insulin at 37 °C in a humidified atmosphere containing 5% CO_2_ [44].

The main reagents used in this study included Lindane (γ-HCH; Sigma-Aldrich, St. Louis, MO, USA, Cat. No. 45603), CCK-8 cell viability assay kit (Beyotime Biotechnology, Shanghai, China, Cat. No. C0038), DCFH-DA reactive oxygen species detection kit (Beyotime Biotechnology, Cat. No. S0033S), and Annexin V-FITC/PI apoptosis detection kit (LiankeBio, Hangzhou, China, Cat. No. AP101-100). The major instruments used in this study included a Multiskan FC microplate reader (Thermo Fisher Scientific, Waltham, MA, USA), BD FACSCanto II flow cytometer (BD Biosciences, San Jose, CA, USA), IX73 inverted fluorescence microscope equipped with DP74 digital camera (Olympus, Tokyo, Japan), QuantStudio 5 real-time PCR system (Applied Biosystems, ABI, Foster City, CA, USA), ChemiDoc XRS + chemiluminescence imaging system (Bio-Rad, Hercules, CA, USA), and Forma Steri-Cycle i160 CO_2_ incubator (Thermo Fisher Scientific, Waltham, MA, USA). Corning (NY, USA)/NEST(Wuxi, China) 96-well plates (#3599) and 6-well plates (#3516) were used for cell culture and experimental assays.

### 4.15. CCK-8 Assay for Cell Viability

The experimental groups included a control group (Control), a low-dose Lindane treatment group (Lindane-L), and a high-dose Lindane treatment group (Lindane-H), with three independent biological replicates performed for each group. The effects of Lindane exposure were evaluated using functional assays and molecular analyses, including assessments of cell viability, oxidative stress, apoptosis, and alterations in key target gene and protein expression levels.

The CCK-8 assay was performed to evaluate the effects of Lindane exposure on cell viability. Cells in the logarithmic growth phase were seeded into 96-well plates at a density of 1 × 10^4^ cells/well, with 100 μL of culture medium added to each well. After cell adhesion, cells were treated with different concentrations of Lindane (0, 5, 10, 20, and 40 μM). After 24 h of Lindane exposure, CCK-8 reagent was added, and the cells were further incubated according to the manufacturer’s instructions. The absorbance at 450 nm was measured using a microplate reader. Relative cell viability was calculated based on the absorbance values, and appropriate Lindane concentrations were selected for subsequent experiments [45].

### 4.16. Reactive Oxygen Species (ROS) Detection

Intracellular reactive oxygen species (ROS) levels following Lindane exposure were measured using the DCFH-DA fluorescent probe. Cells were treated with different concentrations of Lindane for 24 h and subsequently incubated with the DCFH-DA probe according to the manufacturer’s protocol. The fluorescence intensity of cells was measured using flow cytometry, and intracellular ROS accumulation was evaluated based on changes in fluorescence signals [46].

### 4.17. Detection of Lindane-Induced Cell Apoptosis by Annexin V-FITC/PI Double Staining

To evaluate the effects of Lindane exposure on cell apoptosis, Annexin V-FITC/PI double staining combined with flow cytometry was performed [47]. After Lindane exposure, both adherent and suspended cells were collected and washed with PBS, followed by staining according to the instructions provided in the apoptosis detection kit. The percentage of apoptotic cells was measured by flow cytometry, and early and late apoptotic cell populations were identified according to Annexin V and PI staining patterns. All experiments were performed with three independent biological replicates.

### 4.18. Quantitative Real-Time PCR Analysis of Key Gene Expression

To investigate the effects of Lindane exposure on the mRNA expression levels of key genes, quantitative real-time PCR (qRT-PCR) was performed to detect NR3C2, TGM2, and ATAD2 expression. After Lindane exposure, total RNA was extracted from cells and reverse-transcribed into cDNA. qRT-PCR was subsequently performed using a SYBR Green-based detection system. Relative gene expression levels were calculated using the 2^−ΔΔCt^ method, with GAPDH used as the internal reference gene for normalization. All experiments were performed with three independent biological replicates, and each sample contained 2–3 technical replicates [48].

### 4.19. Western Blot Analysis of Protein Expression

To further evaluate the effects of Lindane exposure on apoptosis-associated and target proteins, Western blot analysis was performed to evaluate protein expression changes. After Lindane exposure, cells were collected and lysed, and protein concentrations were determined using the BCA method. Equal amounts of protein were separated by SDS-PAGE and transferred onto PVDF membranes. After blocking, the membranes were incubated with primary antibodies against target proteins, including NR3C2, p53, Bcl-2, and cleaved caspase-3, followed by incubation with corresponding secondary antibodies. Protein signals were detected using an ECL chemiluminescence system, and densitometric analysis was performed to quantify relative protein expression levels [49].

### 4.20. Statistical Analysis

Statistical analyses were performed using GraphPad Prism 9.5.1. Data are presented as mean ± standard deviation (SD). All in vitro experiments were performed with three independent biological replicates. For comparisons among multiple groups, one-way analysis of variance (ANOVA) was performed. Dunnett’s multiple comparisons test was used for CCK-8 assays to compare Lindane-treated groups with the control group. Tukey’s multiple comparisons test was used for qRT-PCR, ROS fluorescence, flow cytometry, and Western blot analyses. A *p* value < 0.05 was considered statistically significant.

## 5. Conclusions

This study provides insights into the potential molecular mechanisms underlying Lindane-associated breast cancer responses through an integrated framework combining network toxicology, machine learning, molecular modelling, and in vitro validation. A total of 28 overlapping molecular targets were identified between Lindane exposure and breast cancer, with enrichment analyses suggesting their involvement in lipid metabolism-related processes, mitochondrial functions, sphingolipid metabolism, and p53-associated pathways. Machine learning approaches identified five candidate hub genes, including NR3C2, AR, TGM2, ATAD2, and P2RY2. Among these candidates, NR3C2 exhibited the strongest diagnostic performance and showed consistent downregulation across independent GEO and TCGA datasets. Molecular docking and molecular dynamics simulations provided structural evidence supporting a potential interaction between Lindane and NR3C2. Cellular experiments further demonstrated that Lindane exposure reduced cell viability, increased intracellular ROS accumulation, and promoted apoptosis-related cellular responses. These effects were accompanied by decreased NR3C2 expression, increased p53, Bax, and cleaved caspase-3 expression, and reduced Bcl-2 expression. Collectively, these findings suggest that oxidative stress and apoptosis-related responses may contribute to Lindane-associated cellular effects, with NR3C2 representing a potential molecular node within this regulatory network. Overall, this study establishes a multi-level analytical framework integrating computational prediction and experimental validation to investigate environmental pollutant-associated cancer mechanisms. The consistent evidence obtained from bioinformatics analysis, machine learning screening, molecular modelling, and cellular experiments supports the potential involvement of NR3C2 in Lindane-associated breast cancer responses. Further studies using larger clinical cohorts and functional approaches, including gene manipulation and pathway intervention experiments, are required to clarify the precise mechanistic role of NR3C2.

## Figures and Tables

**Table 1:** Detailed information on GEO datasets used in the study.

DataSets	Platforms	Sample Size		Organism	Tissue	Attribute
		Control	BC			
GSE22820	GPL6480	10	176	Homo sapiens	breast	Training
GSE26304	GPL6848	6	109	Homo sapiens	breast	Training
GSE57297	GPL17077	7	25	Homo sapiens	breast	Validation
GSE139038	GPL27630	24	41	Homo sapiens	breast	Validation
GSE205185	GPL21185	5	17	Homo sapiens	breast	Validation

## Data Availability

The publicly available transcriptomic datasets analyzed in this study are available from the NCBI Gene Expression Omnibus (https://www.ncbi.nlm.nih.gov/geo/, accessed on 15 March 2026) under accession numbers GSE22820, GSE26304, GSE57297, GSE13903 and GSE205185. The original contributions generated in this study are included in the Supplementary Materials. Additional processed data, analysis outputs, molecular-modeling files, and uncropped experimental images are available from the corresponding authors upon reasonable request.

## Supplementary Materials

The following supporting information can be downloaded at: https://www.mdpi.com/article/10.3390/ijms27209056/s1.

## Abbreviations

The following abbreviations are used in this manuscript:

BC	Breast cancer
EDCs	Endocrine-disrupting chemicals
ROS	Reactive oxygen species
SHAP	Shapley Additive Explanations
WGCNA	Weighted gene co-expression network analysis
TOM	Topological Overlap Matrix
GO	Gene Ontology
KEGG	Kyoto Encyclopedia of Genes and Genomes
BP	Biological Process
CC	Cellular Component
MF	Molecular Function
Stepglm	Stepwise generalized linear model
XGBoost	Extreme gradient boosting
RF	Random forest
plsRglm	Elastic network, partial least squares generalized linear model
GBM	Generalized boosted regression model
LDA	Linear discriminant analysis
SVM	Support vector machine
AUC	Area under the receiver operating characteristic curve
GSVA	Gene set variation analysis
TCGA	The Cancer Genome Atlas
PDB	Protein Data Bank
MD	Molecular dynamics
RMSD	Root mean square deviation
RMSF	Root mean square fluctuation
RG	Radius of gyration
SASA	Solvent-accessible surface area
MM/GBSA	Molecular mechanics/generalized Born surface area
FBS	fetal bovine serum
qRT-PCR	quantitative real-time PCR
ROC	Receiver operating characteristic
GPI	Glycosylphosphatidylinositol
HCM	Hypertrophic cardiomyopathy
POPs	Persistent organic pollutants
